# Electric Field-Responsive Gold Nanoantennas for the Induction of a Locoregional Tumor pH Change Using Electrolytic Ablation Therapy

**DOI:** 10.1021/acsnano.4c03610

**Published:** 2024-07-08

**Authors:** Ara Joe, Panchanathan Manivasagan, Jong Kook Park, Hyo-Won Han, Sun-Hwa Seo, Thavasyappan Thambi, Vu Hoang Giang Phan, Soon Ah Kang, João Conde, Eue-Soon Jang

**Affiliations:** † Department of Applied Chemistry, Kumoh National Institute of Technology, Daehak-ro 61, Gumi, Gyeongbuk 39177, Republic of Korea; ‡ Department of Convergence Technology, Graduate School of Venture, Hoseo University, Seoul 06724, Republic of Korea; § Graduate School of Biotechnology, College of Life Sciences, Kyung Hee University, Yongin-si, Gyeonggi-do 17104, Republic of Korea; ∥ Biomaterials and Nanotechnology Research Group, Faculty of Applied Sciences, Ton Duc Thang University, Ho Chi Minh City 70000, Vietnam; ⊥ ToxOmics, NOVA Medical School, Faculdade de Ciências Médicas, NMS|FCM, Universidade NOVA de Lisboa, Lisboa 1169-056, Portugal

**Keywords:** gold nanorods, electrolytic ablation, localized electric field, colorectal cancer, Pt electrodes, locoregional pH

## Abstract

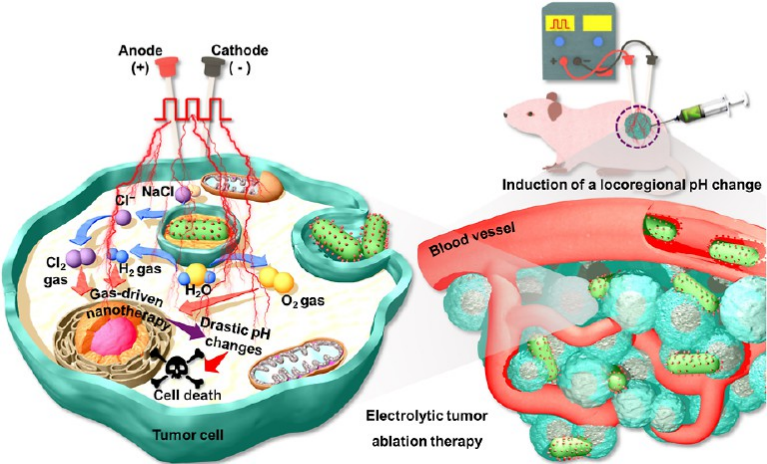

Electrolytic ablation (EA) is a burgeoning treatment for solid tumors, in which electrical energy catalyzes a chemical reaction to generate reactive species that can eradicate cancer cells. However, the application of this technique has been constrained owing to the limited spatial effectiveness and complexity of the electrode designs. Therefore, the incorporation of nanotechnology into EA is anticipated to be a significant improvement. Herein, we present a therapeutic approach based on difructose dianhydride IV-conjugated polyethylenimine-polyethylene glycol-modified gold nanorods as electric nanoantennas and nanoelectrocatalysts for EA. We demonstrate that square-wave direct current (DC) fields trigger a reaction between water molecules and chloride ions on the gold nanorod surface, generating electrolytic products including hydrogen, oxygen, and chlorine gases near the electrodes, changing the pH, and inducing cell death. These electric nanoantennas showed significant efficacy in treating colorectal cancer both in vitro and in vivo after DC treatment. These findings clearly indicate that gold nanoantennas enhance the effectiveness of EA by creating a localized electric field and catalyzing electrolytic reactions for the induction of locoregional pH changes within the tumor. By overcoming the limitations of traditional EA and offering an enhanced level of tumor specificity and control, this nanotechnology-integrated approach advances further innovations in cancer therapies.

Colorectal cancer, ranking third in prevalence and lethality worldwide, presents profound public health concerns on a global scale.^1^ Typically, treatment of advanced-stage solid tumors entails surgery complemented by chemotherapy and radiotherapy as auxiliary measures.^2^ To some extent, these conventional treatment modalities can manage disease progression in advanced solid tumors and metastatic conditions.^3^ However, they come with a suite of clinical limitations, including toxicity, side effects, and potential resistance.^4^ The ongoing challenges of these therapies underscore the urgent need for innovative therapeutic strategies for solid-tumor ablation.^5^

Emerging from the latest advancements in oncology, ablation has shown promise as an alternative method for eradicating advanced-stage solid tumors.^6^ Ablation therapies work by applying localized energy to the tumor site using either thermal or nonthermal energy sources.^7^ Thermal-based ablation methods, such as phototherapy, interstitial laser therapy, microwave ablation, radiofrequency ablation, and high-intensity focused ultrasound, which leverage thermal energy, have shown efficacy in disrupting tumors at various stages by raising temperatures and inducing hyperthermia in tumor cells.^8^ However, the thermal ablation methods have intrinsic limitations. For instance, they can affect the immediate tumor environment, particularly when major blood vessels or bile ducts traverse the target tumors, causing potential harm to surrounding healthy tissues.^7^ Consequently, nonthermal ablation methods have emerged as more efficient and safer alternatives.

One of the most promising techniques is electrolytic ablation (EA), also known as electrochemical therapy.^5^ EA operates based on the principles of electrochemistry to eliminate solid tumors.^9^ The procedure involves the local insertion of two platinum electrodes into the tumor, followed by the application of a low-voltage, square-wave, direct-current electric field.^10^ The electrical energy then transforms into chemical energy at the electrode sites, generating a series of gases that create drastic pH variations in the local environment, thereby efficiently annihilating the tumor.^11^ Clinical trials have demonstrated promising outcomes, with EA offering a host of advantages, such as simplicity, safety, low cost, and precision, while minimizing side effects.^12^ Despite these benefits, EA faces challenges pertaining to electrode configuration and effective volume, necessitating further advancements in the technique.^10,13^

Recent breakthroughs in nanotechnology have provided exciting solutions for these challenges.^10^ EA therapies are now leveraging nanomaterials, particularly noble metal nanoparticles such as gold nanorods (GNRs), which function as electric nanoantennas and nanoelectrocatalysts.^14^ GNRs possess superior electrocatalytic capabilities, biocompatibility, and stability, rendering them effective for EA.^15^ However, the synthesis of GNRs involves the surfactant cetyltrimethylammonium bromide (CTAB), which is considerable cytotoxicity issue.^16^ To overcome this, alternative approaches, such as polymer multilayer surface coatings, have been employed to alleviate cytotoxic effects.^17^

The use of biocompatible polymers, specifically thiol polyethylene glycol (PEG) and polyethylenimine (PEI), is viewed as an optimal solution for GNR surface modification.^18^ These polymers offer a range of advantages, including excellent biocompatibility, chemical stability, solubility, and nontoxicity.^19^ PEI–PEG-GNRs can be produced by combining these polymers with the GNR core.

In the context of surface modification, difructose dianhydride IV (DFA IV) emerges as a promising candidate.^20^ DFA IV is a natural prebiotic with multiple health-promoting properties.^21^ Its main advantage lies in its nondigestible nature and capacity to enhance the absorption of mineral ions.^22^ This characteristic can potentiate the transfection of GNRs into colorectal cancer cells, thereby advancing innovative therapeutic strategies.^22^

The incorporation of DFA IV into PEI–PEG-GNRs has garnered attention owing to its potential use as an electric nanoantenna in EA therapy. These DFA IV-conjugated PEI–PEG-GNRs possess promising attributes such as electrocatalytic capability, biocompatibility, conductivity, and stability. Thus, we propose a conceptual approach for electrolytic tumor ablation therapy using biocompatible DFA IV-PEI-PEG-GNRs (Figure 1). By advancing this promising therapy, we aim to address the unmet needs in colorectal cancer treatment and potentially improve the outcomes for patients worldwide.

**Figure 1:**
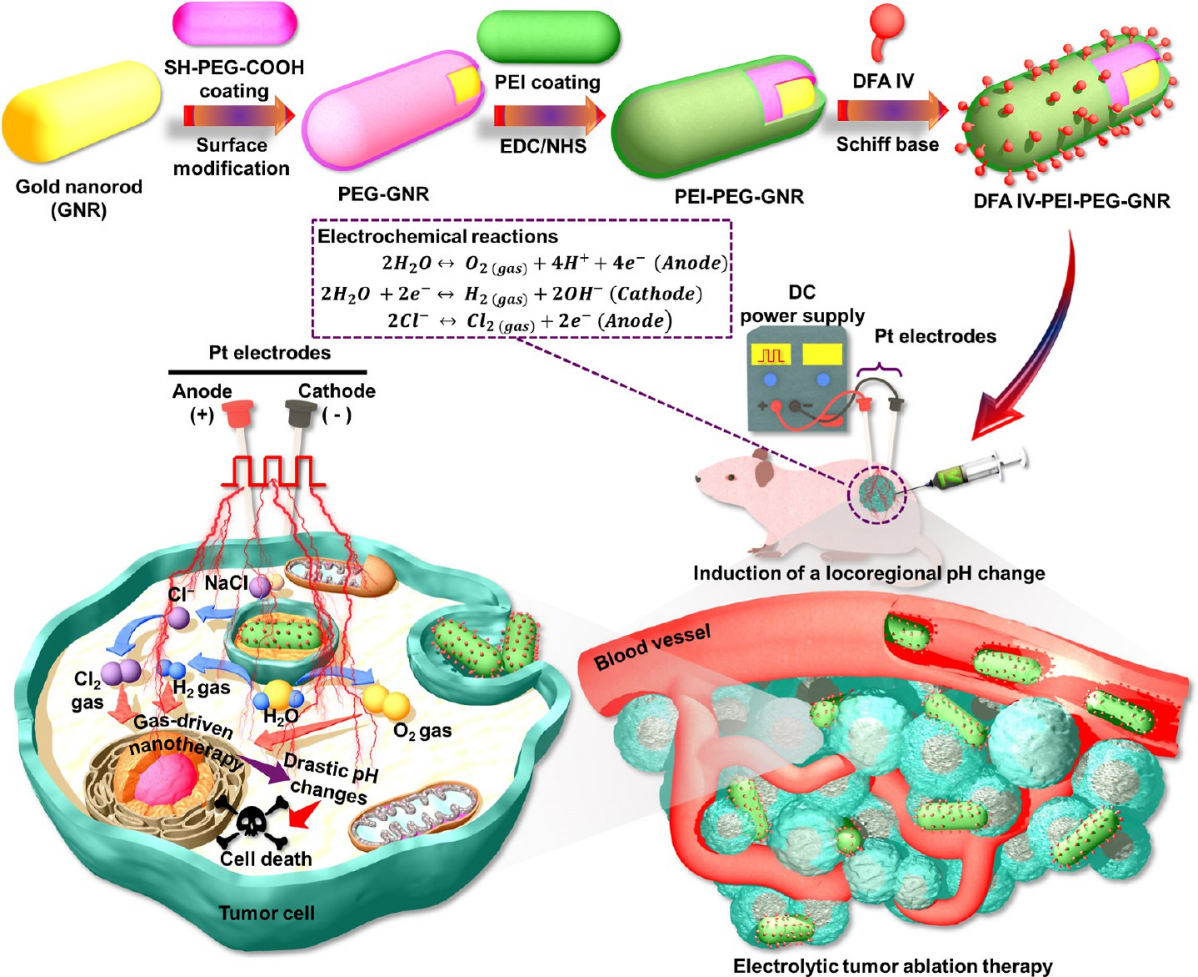
Schematic of the fabrication process of DFA IV-PEI–PEG-GNRs and the therapeutic mechanism of electrolytic tumor ablation therapy. GNRs were synthesized using a facile seed-mediated growth technique, and the GNR surface was modified with PEG/PEI. DFA IV was conjugated to the surface of PEI–PEG-GNRs using a Schiff base reaction to produce DFA IV-PEI–PEG-GNRs. When a direct current (DC) is applied, the DFA IV-PEI–PEG-GNRs accelerate the dissociation of water molecules and produce a host of electrochemical reaction products such as oxygen (O_2_), hydrogen (H_2_), and chlorine gas (Cl_2_). These reaction products cause significant changes in the pH environment of tumor tissues, leading to an extremely high nanoelectrocatalytic efficiency, which is lethal to tumor cells.

## Results and Discussion

### Rational Design and Characterization of Electric Field-Responsive Gold Nanoantennas

Gold nanorods (GNRs) were synthesized as electric nanoantennas using a facile seed-mediated growth technique in the presence of CTAB.^23^ CTAB-capped GNRs are not useable in their current forms because of their cytotoxicity.^16^ Therefore, a biocompatible dual polymer, such as PEG/PEI multilayer coating, is required to cover the GNR core surface. Thiol-PEG-carboxyl (SH-PEG-COOH) is one of the most suitable candidates for the surface modification of GNRs, enhancing their stability and biocompatibility.^19^ For the conjugation of thiol-PEG-carboxyl ligands to CTAB-capped GNRs, PEG-modified GNRs (PEG-GNRs) were prepared according to a previously reported methodology,^24^ where GNRs were covalently conjugated by forming PEG on the surface containing a thiol functional group at one end and a carboxyl group at the other, implying the formation of carboxyl-PEG-GNRs (PEG-GNRs) based on classic covalent gold–thiol bond chemistry.^25^ PEI was used as the amine source because of its high density of amine groups and easily accessible primary, secondary, and tertiary amine sites at each chain end.^26^ The primary PEI amine was covalently coupled with the carboxyl group of the PEG-GNRs using EDC/NHS chemistry to enhance biocompatibility. DFA IV is a cyclic, nondigestible, and versatile disaccharide with multiple physiological functions. It may serve as a potential prebiotic candidate for the treatment and prevention of colorectal cancer, anemia, and osteoporosis.^21^ DFA IV was conjugated to the surface of the PEI–PEG-GNRs using the Schiff base reaction (Figure S1) to produce DFA IV-PEI–PEG-GNRs (Figures S2 and S3) for electrolytic tumor ablation therapy.

**Figure 2:**
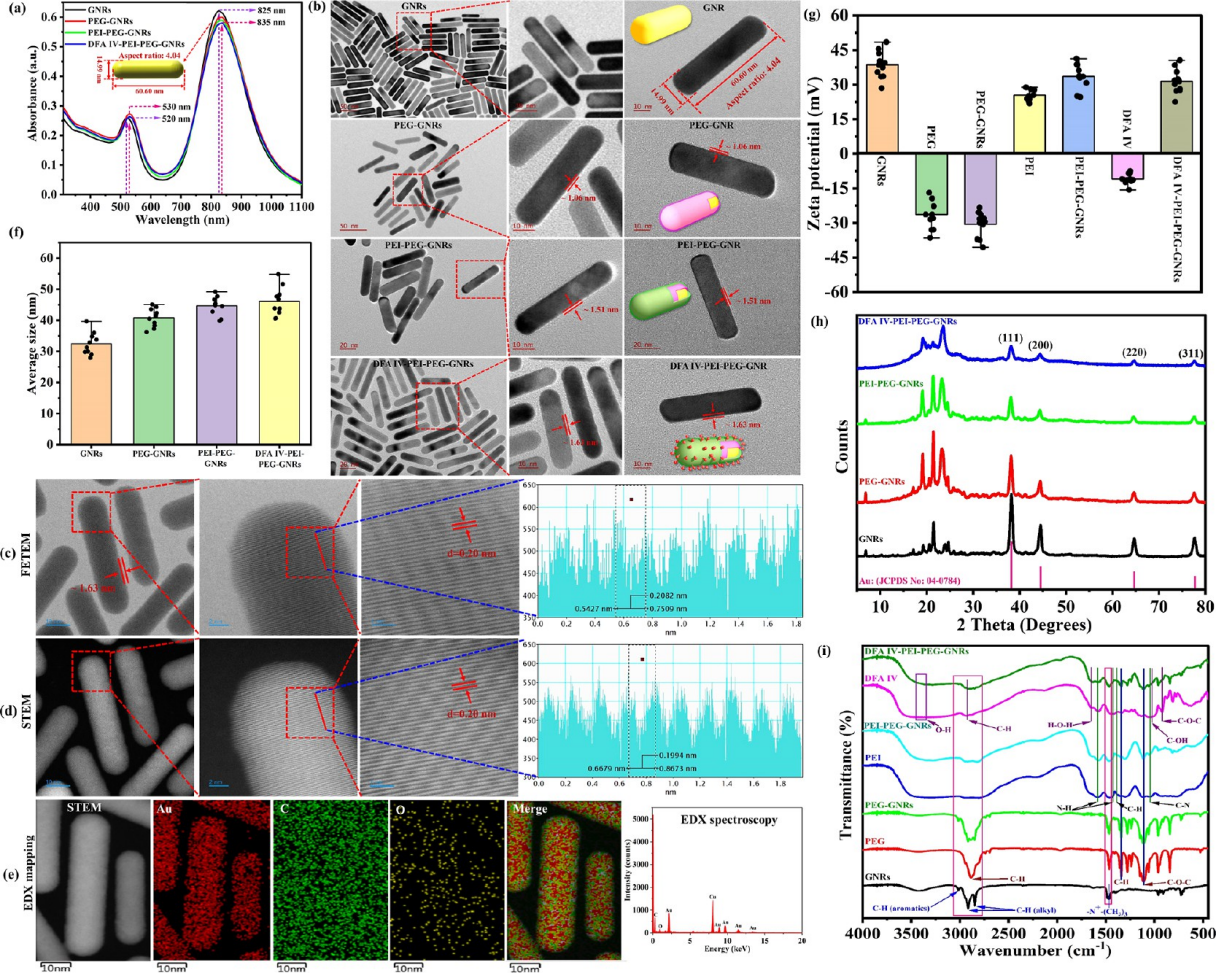
(a) UV–NIR spectra of GNRs, PEG-GNRs, PEI–PEG-GNRs, and DFA IV-PEI–PEG-GNRs. (b) TEM images of GNRs, PEG-GNRs, PEI–PEG-GNRs, and DFA IV-PEI–PEG-GNRs. (c) High-magnification FETEM images showing the lattice fringes and lattice fringe profiles of the DFA IV-PEI–PEG-GNRs. (d) High-magnification STEM images to show the lattice fringes and lattice fringe profile of DFA IV-PEI–PEG-GNRs. (e) EDX elemental mapping and spectroscopy of the DFA IV-PEI–PEG-GNRs. (f) DLS analysis of GNRs, PEG-GNRs, PEI–PEG-GNRs, and DFA IV-PEI–PEG-GNRs. (g) ζ-potentials of GNRs, PEG, PEG-GNRs, PEI, PEI–PEG-GNRs, DFA IV, and DFA IV-PEI–PEG-GNRs. (h) XRD patterns of the GNRs, PEG-GNRs, PEI–PEG-GNRs, and DFA IV-PEI–PEG-GNRs. (i) FTIR spectra of GNRs, PEG, PEG-GNRs, PEI, PEI–PEG-GNRs, DFA IV, and DFA IV-PEI–PEG-GNRs.

The UV–vis–NIR spectra of the GNRs, PEG-GNRs, PEI–PEG-GNRs, and DFA IV-PEI–PEG-GNRs are shown in Figure 2a. GNRs exhibit two distinct surface plasmon resonance (SPR) bands: a weak transverse SPR band at 530 nm and a strong longitudinal SPR (LSPR) band at 825 nm, which demonstrates their capability to absorb light at 823 nm, consistent with their ability to act as electric nanoantennas. The LSPR absorption peak at 800–840 nm represents a GNR aspect ratio (AR) of 4.04.^17^ After surface modification, a slight red shift was observed in the LSPR band at 835 nm for the PEG-GNRs, PEI–PEG-GNRs, and DFA IV-PEI–PEG-GNRs, probably because of their coating with PEI and PEG. Similar findings have been reported for PEG coatings that enhance the LSPR band shift.^27^ Transmission electron microscopy (TEM) imaging of all four GNR types (Figure 2b) shows that their morphology is uniform, with well-defined rod-like structures with a mean length of 60.60 ± 2.7 nm and mean width of 14.99 ± 3.35 nm, yielding an AR of 4.04. PEG conjugation formed a faint layer surrounding each GNR with a thickness of ∼1.06 nm, implying that the GNRs were entirely covered with PEG. After coating the PEG-GNR surface with PEI, we observed a thin, uniform layer of PEI with a thickness of ∼1.51 nm. PEI–PEG-GNRs conjugated with DFA IV exhibited a uniform morphology with a thickness of ∼1.63 nm. DFA IV-PEI–PEG-GNR morphology was further characterized using field-emission TEM and scanning transmission electron microscopy (FETEM and STEM, respectively), revealing unchanged and well-defined rod-like structures after surface modification (Figure 2c), with a single-crystalline nature over their entire lengths, and clear lattice fringes (0.20 nm that could be assigned to the (1 1 1) planes of face-centered cubic (fcc) gold (Au) (Figure 2d). The formation of DFA IV-PEI–PEG-GNRs was further characterized by energy-dispersive X-ray (EDX) mapping and spectroscopy (Figure 2e), revealing that Au, C, and O were uniformly distributed throughout, demonstrating that different functional components were successfully conjugated to the surface of the GNRs.

The average hydrodynamic size and distribution of all four GNR types were analyzed using dynamic light scattering (DLS) (Figure 2f). The mean for GNRs was 32.48 ± 1.62 nm, indicating a highly monodisperse nature and uniformly distributed rod-like structures. GNR size was increased by functionalization with PEG and PEI to 40.82 ± 2.04 and 44.63 ± 2.23 nm, respectively, confirming the surface modifications. The mean hydrodynamic size of DFA IV-PEI–PEG-GNRs was 46.05 ± 2.30 nm. The ζ-potential surface charge (Figure 2g) provides direct evidence for the surface functionalization of GNRs with various functional components. The ζ-potentials of GNRs, PEG, PEG-GNRs, PEI, PEI–PEG-GNRs, DFA IV, and DFA IV-PEI–PEG-GNRs were +38.56 ± 2.02, −26.37 ± 1.31, −30.61 ± 1.53, +25.46 ± 1.27, +33.56 ± 1.67, −10.92 ± 1.54, and +31.48 ± 1.58 mV, respectively, indicating that GNRs functionalized with various functional components.

The crystalline structures and compositions of the GNRs, PEG-GNRs, PEI–PEG-GNRs, and DFA IV-PEI–PEG-GNRs were determined from the X-ray diffraction (XRD) patterns (Figure 2h). The XRD pattern of the GNRs showed four diffraction lines at 2θ = 38.23, 44.41, 64.61, and 77.72°, assigned to the (111), (200), (220), and (311) planes of the fcc structure of metallic Au (JCPDS no. 04-0784), respectively, indicating that the GNRs have a crystalline nature. PEG-GNRS showed two strong reflections at 2θ = 19.13 and 23.52° for PEG and four strong reflections at 2θ = 38.23, 44.41, 64.61, and 77.72° for GNRs, which is consistent with the conjugation of PEG-GNRs. PEI exhibited a wide peak between 2θ = 15 and 30°, but no sharp narrow peaks were observed. PEI–PEG-GNRs exhibited a broad diffraction peak of PEI, two sharp peaks of PEG, and four diffraction peaks of GNRs, indicating a dual-polymer coating such as PEG/PEI. For pure DFA IV, a crystalline peak appeared between 2θ = 10 and 30°, but no sharp peaks were observed. Primary peaks of DFA IV, PEI, PEG, and GNRs were observed for DFA IV-PEI–PEG-GNRs, indicating their crystalline nature.

Figure 2i shows the Fourier-transform infrared (FTIR) spectra of the GNRs, PEG, PEG-GNRs, PEI, PEI–PEG-GNRs, DFA IV, and DFA IV-PEI–PEG-GNRs. GNRs showed major characteristic bands at 3019, 2918, and 2849 cm^–1^, caused by the C–H stretching vibrations of the aromatic and alkyl groups of CTAB. The strong absorption at 1468 cm^–1^ was also characteristic of the N^+^–(CH_3_)_3_ asymmetric stretching vibrations of the CTAB surface bilayer, confirming that CTAB was still negligible on the surface of the GNRs.^28^ PEG was observed at 1119 cm^–1^, which was attributable to C–O–C stretching vibrations. C–H stretching bands corresponding to PEG chains at 1339 and 2883 cm^–1^ were also observed. For the conjugation of PEG on the GNR surfaces (PEG-GNRs), the C–H and N^+^–(CH_3_)_3_ bands corresponding to GNRs at 3019, 2918, 2849, and 1468 cm^–1^ were weakened and disappeared, whereas the C–O–C and C–H bands corresponding to PEG were observed at 1119, 1339 and 2883 cm^–1^, indicating surface coating by PEG. PEI exhibited strong characteristic bands at 1436 and 1591 cm^–1^, associated with the N–H vibrations of the primary and secondary amine groups, respectively. The bands at 1390 and 1050 cm^–1^ correspond to the C–H and C–N stretching vibrations of PEI, respectively. For amine surface functionalization, the primary absorption bands were 1436, 1591, 1390, and 1050 cm^–1^, associated with N–H, C–H, and C–N stretching vibrations, respectively. The major absorption bands of PEG and GNRs also appeared, confirming that both polymers (PEI/PEG) were coated onto the GNR surface. DFA IV showed a characteristic broad peak at 3401 cm^–1^ corresponding to O–H, and also exhibited peaks at 2928, 1649, 1029, and 926 cm^–1^, assigned to C–H stretching, H–O–H scissors, C–OH stretching, and C–O–C stretching vibrations, respectively. After conjugation of DFA IV, a characteristic peak at 1649 cm^–1^ was observed, with a slight shift attributable to C–H and C–O–C stretching vibrations. Primary PEI, PEG, and GNR bands also appeared, indicating DFA IV conjugation. The stability of NPs is a key parameter for biomedical applications. The PEG-GNRs, PEI–PEG-GNRs, and DFA IV-PEI–PEG-GNRs were dispersed in deionized water (DW), phosphate-buffered saline (PBS), and Dulbecco’s modified Eagle mediums (DMEM) without phenol red with 10% fetal bovine serum (FBS) for 7 days, and the solutions were analyzed using a UV-2600 spectrometer. The results showed that there were no obvious changes in the UV–vis spectra of these NPs under various conditions, which suggests a highly stable nature (Figure S4).

### Enhanced Stimuli-Activated Electrolytic Capacity

**Figure 3:**
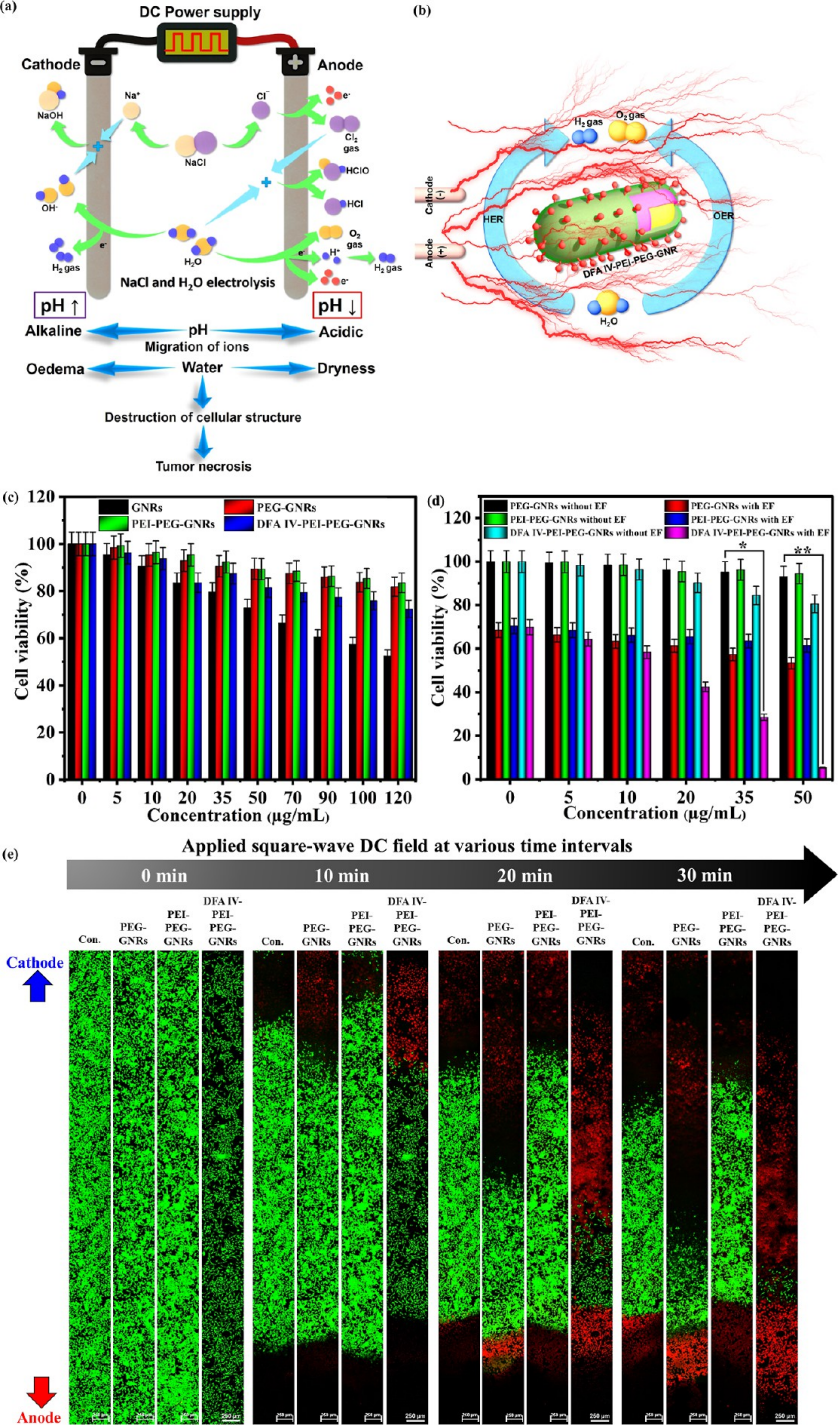
(a) Electrochemical reactions occurring at the cathode and anode during the electrolytic process. (b) Schematic illustration of DFA IV-PEI–PEG-GNRs for electrochemical reactions in water (HER and OER). (c) Relative viability of CT-26 cells after treatment with GNRs, PEG-GNRs, PEI–PEG-GNRs, and DFA IV-PEI–PEG-GNRs at various concentrations (0–120 μg/mL) for 24 h. (d) Relative viability of CT-26 cells incubated with various concentrations of PEG-GNRs, PEI–PEG-GNRs, and DFA IV-PEI–PEG-GNRs with or without a square-wave DC field for 30 min *p* values: *p < 0.05, **p < 0.01. (e) Merged confocal fluorescence images in the tile scan mode of live (green) and dead (red) cells stained with calcein-AM and PI after treatment with PEG-GNRs, PEI–PEG-GNRs, and DFA IV-PEI–PEG-GNRs with square-wave DC field for various time intervals (10× magnification; scale bar: 250 μm).

EA is a promising technique that electrochemically destroys cancer cells and tumor tissues. The primary electrochemical reactions involved in low-level square-wave DC-induced electrolysis during in situ local pH modulation are shown in Figure 3a. The primary mechanism of electrochemical tumor ablation has been described by Li et al.^29^ Electrochemical reactions occur during electrolysis when two Pt electrodes are injected into cancer cells and tumor tissues, after which the application of a square-wave DC prompts the rapid dissociation of sodium chloride [NaCl, Reaction (Reaction 1)], and the electrolysis of water (H_2_O).^30^
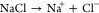
1

The primary effect of electrolysis is the production of hydrogen ions (H^+^, hydronium) and hydroxide ions (OH −) according to the following reactions
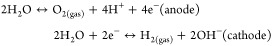


A secondary electrolysis effect was observed in the NaCl solution (Reaction 1), ionized into sodium (Na^+^) and chloride (Cl^–^) ions, which move toward the cathode and anode, respectively. The relevant reactions are as follows
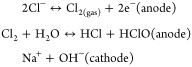


EA of target cancer cells and tumor tissues is initiated by electroosmotic dehydration due to water migration from the anode to the cathode, drying the tissue around the anode, and causing edema at the cathode.^31^ The concentrations of Na^+^ and potassium (K^+^) ions simultaneously increased near the cathode, where alkalinity also increased, changing the hemoglobin to alkaline hemin surrounding the cathode, and the concentration of chloride (Cl^–^) ions increased around the anode, changing the hemoglobin to acidic hemin.^29^ Na^+^ and Cl^–^ ions react with tissue water to generate sodium hydroxide (NaOH) and hydrogen (H_2_) gas at the cathode and hydrochloric acid (HCl), hypochlorous acid (HClO), oxygen (O_2_) gas, and chlorine (Cl_2_) gas at the anode according to the reactions mentioned above.^32^ Among the gases generated electrochemically, Cl_2_ gas is a strong oxidant that damages and bleaches surrounding tissues. NaOH is also toxic but to a lesser extent. H_2_ gas can cause local cavitation in tumor tissues, and O_2_ gas can increase acidity because the generated protons (H^+^) promptly undergo hydration to produce hydronium (H_3_O^+^) ions.^11^ Thus, tumor tissues around the anode become acidic (pH < 6.0), whereas those around the cathode become alkaline (pH > 9.0), causing cell death in the vicinity of.^9^ Electrochemical tumor ablation therapy is an emerging external stimuli-activated therapeutic strategy that can efficiently achieve satisfactory treatment outcomes with fewer side effects.^5,10^ Recently, noble metal NPs, such as GNRs, have been widely recognized as nanoelectrocatalysts for electrochemical tumor ablation therapy because of their superior catalytic ability, biocompatibility, versatility, stability, conductivity, and selectivity.^33^ GNRs can generate H_2_ gas under a square-wave DC electric field, making them one of the most effective electrocatalysts for the hydrogen evolution reaction (HER) and oxygen evolution reaction (OER) (Figure 3b).^34^ HER occurs at the cathode during electrolysis, whereas the OER occurs at the anode.
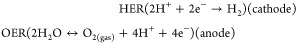


To further enhance the therapeutic effect, a combination of Pt electrodes and GNRs is highly desirable for enhancing electrochemical reactions, implying that a synergistic effect can successfully cause pH changes near the electrodes, resulting in cell death.^35^ DFA IV-PEI–PEG-GNRs accelerated the dissociation of NaCl and water molecules in tumor tissues under a square-wave DC electric field. The electrolysis reaction products (H_2_, H^+^, NaOH, Cl_2_, HClO, HCl, O_2_, etc.) around the electrodes can induce significant pH changes in tumor tissues, leading to an extremely high nanoelectrocatalytic efficiency for tumor abrogation.

### High Biocompatibility and Effective Cellular Uptake of Gold Nanoantennas

First, to measure the cytotoxicity in cultured cells, we conducted an 3-(4,5-dimethylthiazol-2-yl)-2,5-diphenyltetrazolium bromide (MTT) viability assay on CT-26 cells treated with GNRs, PEG-GNRs, PEI–PEG-GNRs, and DFA IV-PEI–PEG-GNRs (Figure 3c). Cells were incubated with the four different types of GNRs at various concentrations (0–120 μg/mL) for 24 h. No apparent toxicity was observed with PEG-GNRs, PEI–PEG-GNRs, or DFA IV-PEI–PEG-GNRs concentrations up to 120 μg/mL, indicating the high biocompatibility of the NPs. However, GNRs demonstrated cytotoxicity owing to the presence of surface-bound CTAB.

The level of gold (Au) internalization in CT-26 cells was measured using atomic absorption spectroscopy (AAS) (Figure S5). The cellular uptake of PEG-GNRs, PEI–PEG-GNRs, and DFA IV-PEI–PEG-GNRs (all 50 μg/mL) in CT-26 cells gradually increased over time. The Au content of DFA IV-PEI–PEG-GNRs was 69.9 ± 3.49 pg/cell at 12 h, compared to PEG-GNRs (22.47 ± 1.12 pg/cell) and PEI–PEG-GNRs (43.47 ± 2.17 pg/cell), implying that DFA IV as a prebiotic can specifically target CT-26 cells for the treatment and prevention of colorectal cancer. Additionally, we used dark-field inverted microscopy to visualize the distribution of PEG-GNRs, PEI–PEG-GNRs, and DFA IV-PEI–PEG-GNRs (all at 50 μg/mL) in CT-26 cells, and evaluated their specific targeting efficacy (Figure S6). The yellow-orange color indicates specific targeting, whereas the endogenous scattering in the control cells is represented by a bluish-gray color.^36^ DFA IV-PEI–PEG-GNRs displayed a strong yellow-orange color, indicating a significantly higher therapeutic targeting efficacy than PEG-GNRs and PEI–PEG-GNRs.

### In Vitro Gas-Driven Electrolytic Ablation Therapy

To evaluate the therapeutic effects of EA on tumor cells, CT-26 cells were treated with PEG-GNRs, PEI–PEG-GNRs, and DFA IV-PEI–PEG-GNRs at concentrations ranging from 0 to 50 μg/mL for 12 h. The cells were then either exposed or not exposed to square-wave DC treatment of 4.3 V/cm and 1.0 mA at two Pt electrodes for 30 min. Cell viability was evaluated after 24 h. As shown in Figure 3d, DFA IV-PEI–PEG-GNRs (50 μg/mL) demonstrated cytotoxicity owing to greater pH changes near the electrodes compared to the other groups, suggesting that DFA IV-PEI–PEG-GNRs exert cytotoxic effects under an electric field.

EA was further assessed in vitro using confocal laser scanning microscopy (CLSM) in the tile scan mode of live–dead cell staining (Figure 3e). CT-26 cells were treated with 50 μg/mL PEG-GNRs, PEI–PEG-GNRs, and DFA IV-PEI–PEG-GNRs for 12 h and then exposed to a square-wave DC field for 0, 10, 20, and 30 min. The cells were then costained with calcein-AM and propidium iodide (PI) for 30 min, resulting in green and red fluorescence. Cells treated with DFA IV-PEI–PEG-GNRs were efficiently killed after 30 min of exposure to the DC field, which was associated with a strong red fluorescence because cell death was significantly higher from the cathode to the intermediate region compared to the anode. The wide range of tile scan images with multiple fluorescence frames allowed visualization of the entire cell culture area affected by the NPs and DC treatments. In contrast, the control, PEG-GNRs, and PEI–PEG-GNRs exhibited strong green fluorescence, almost identical to the middle region, and showed negligible cell death near the cathode and anode.^10^

**Figure 4:**
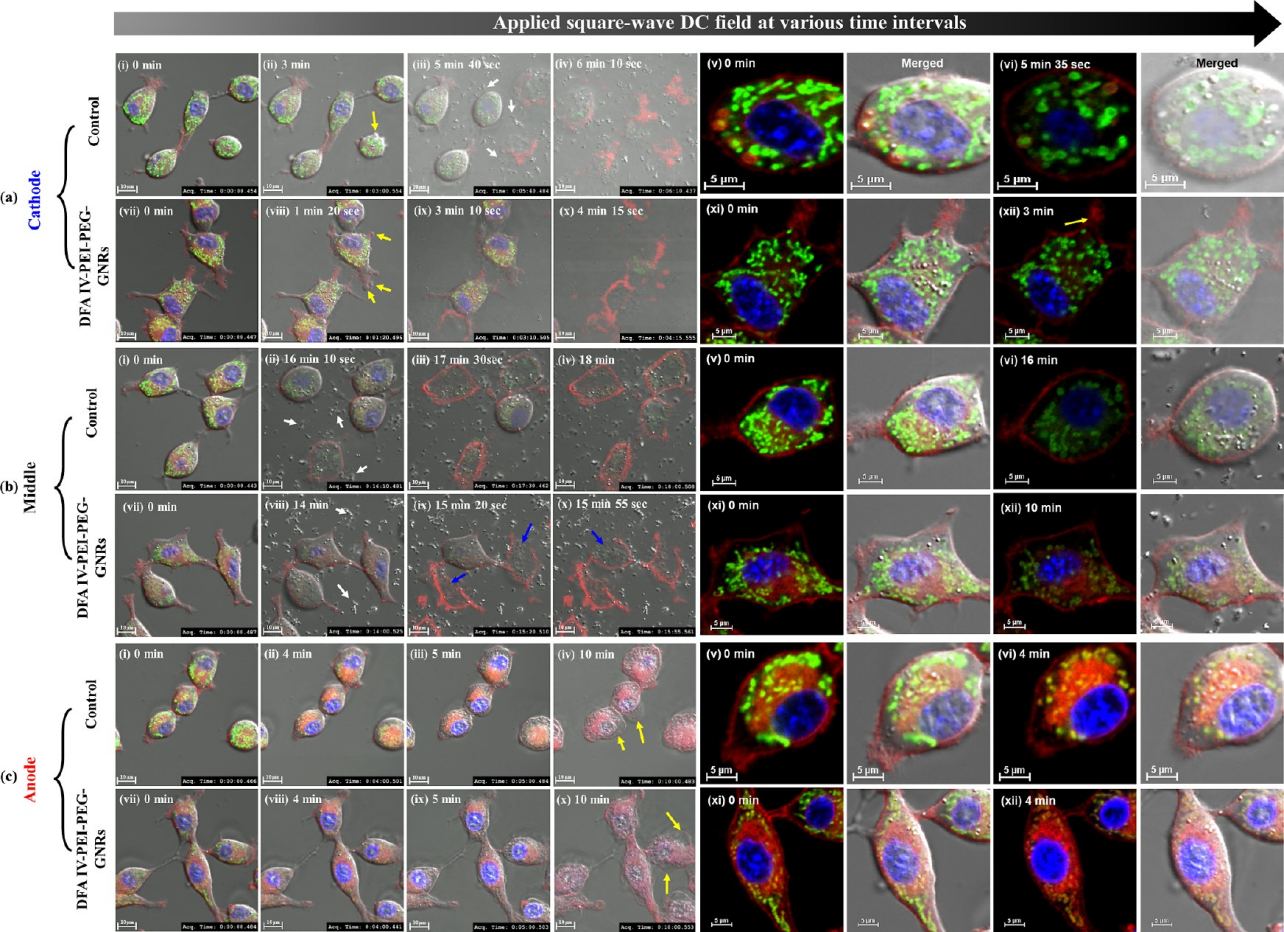
Merged images of confocal fluorescence images and bright field images of CT-26 cells treated without (control) or with DFA IV-PEI–PEG-GNRs at the cathode (a), middle (b), and anode (c) regions under square-wave DC treatment for various time intervals (60× magnification; scale bar: 10 and 5 μm). Cells were stained with Hoechst 33342, MitoTracker Green, ER-Tracker Red, and CellMask Deep Red Plasma Membrane for 30 min to stain the nuclei, mitochondria, ER, and plasma membrane. Yellow arrows indicate membrane blebs. White arrows indicate the migration of released organelle fragments. Blue arrows indicate cell swelling and bursting.

The EA of the DFA IV-PEI–PEG-GNRs was further assessed using multiple staining and CLSM. CT-26 cells were incubated with PBS (100 μL) and DFA IV-PEI–PEG-GNRs (50 μg/mL) for 12 h and subjected to different intervals of square-wave DC field treatment. Cells were stained with Hoechst 33342, MitoTracker Green, endoplasmic reticulum (ER)-Tracker Red, and CellMask Deep Red plasma membrane for 30 min to stain the nuclei, mitochondria, ER, and plasma membrane. Figure 4 shows real-time CLSM images of the control and DFA IV-PEI–PEG-GNR-treated cells at the cathode, middle, and anode regions under square-wave DC treatment. At the 3 and 1 min 20 s time points, membrane blebs appeared in the control cells (Videos S1 and S2) and DFA IV-PEI–PEG-GNRs-treated cells (Videos S3 and S4) at the cathode as indicated by the yellow arrows (Figure 4a (ii,viii)), confirming that DFA IV-PEI–PEG-GNRs induce rapid cell death. At 3 and 5 min, the control and DFA IV-PEI–PEG-GNR-treated cells at the cathode exhibited fast bleb formation and cell swelling due to electroosmotic dehydration [Figure 4a (iii,ix)[. Subsequently, both treated cells at the cathode ruptured, and cytoplasmic organelles were released at the 6 and 4 min time points, respectively [Figure 4a (iv,x)]. At the cathode, mitochondrial swelling and nuclear shrinkage were observed in both treated cells before the plasma membrane was destroyed (Figure 4a (v,vi,xi,xii)). At the cathode, the typical necrotic pathway shows plasma membrane rupture through bleb formation and organelle swelling, resulting in cell death. In contrast, in the control (Videos S5 and S6) and DFA IV-PEI–PEG-GNR-treated cells (Videos S7 and S8) in the middle region, the released organelle fragments rapidly migrated from the cathode to the anode (white arrows), which reached the middle region between the electrodes, causing rapid swelling and bursting (blue arrows) due to hypo-osmotic pressure, implying that cell lysis in the middle region was primarily caused by the electrolytic components generated from the cathode (Figure 4b). Simultaneously, both control (Videos S9 and S10) and DFA IV-PEI–PEG-GNR-treated cells (Videos S11 and S12) at the anode became dry, with the formation of large blebs after the DC field was applied for 5 min (Figure 4c (iii,ix)). When the released organelle fragments migrated from the cathode to the anode, the resulting electroosmotically dehydrated CT-26 cells caused cell swelling. Similar results have been reported, showing that the electroosmotic dehydration of cancer cells at the anode could be induced by the migration of protonated hydronium (H_3_O_+_) ions to the cathode.^31^ As a result of these electrochemical reactions, DFA IV-PEI–PEG-GNR-treated cells near the anode and cathode were subjected to acidic and alkaline pH, respectively. Additionally, DFA IV-PEI–PEG-GNRs-treated cells were grown in strong acidic pH [2.5 (Video S13) and 3.4 (Video S14)) and alkaline pH (10.3 (Video S15) and 11.4 (Video S16)] DMEM by the adding 1 M HCl or 1 M NaOH. DFA IV-PEI–PEG-GNR-treated cells were cultured for multiple time intervals and stained, as shown in Figures S7a–d. The fates of CT-26 cells exposed to acidic and alkaline conditions were identical to those observed at the anode and cathode, respectively, implying that electrochemically generated pH changes were the primary reason for cell death caused by EA.^11^

**Figure 5:**
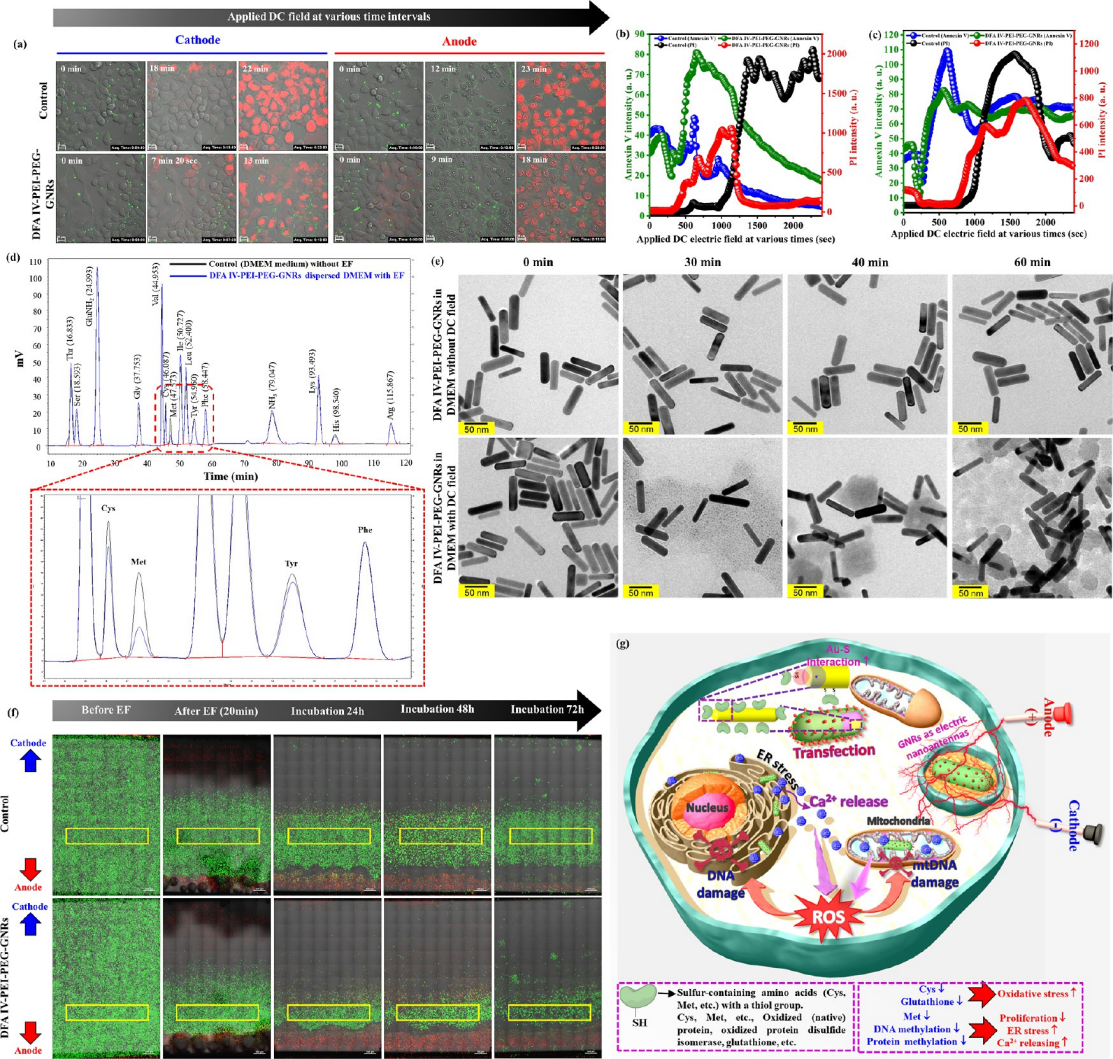
(a) Merged images of confocal fluorescence images and bright field images of CT-26 cell apoptosis induced by PBS (control) and DFA IV-PEI–PEG-GNRs at the cathode and anode with and without square-wave DC field using Annexin V-FITC/PI (60× magnification; scale bar: 20 μm). Fluorescence intensity variations of CT-26 cell apoptosis induced by PBS (control) and DFA IV-PEI–PEG-GNRs at the cathode (b) and anode (c) with/without square-wave DC field using Annexin V-FITC/PI. (d) HPLC chromatogram of the amino acid profile in DMEM only (control) and DFA IV-PEI–PEG-GNRs dispersed in DMEM with or without square-wave DC treatment. (e) TEM images of DFA IV-PEI–PEG-GNRs dispersed in DMEM, with or without a square-wave DC field for 60 min at 37 °C. (f) Merged image of confocal fluorescence images and bright field images in the tile scan mode of live (green) and dead (red) CT-26 cells stained with calcein-AM and PI after treatment with PBS (control) and DFA IV-PEI–PEG-GNRs with or without square-wave DC field for 20 min, followed by incubation for another 24, 48 and 72 h (10× magnification; scale bar: 500 μm). (g) Illustration of the effect of EA on CT-26 cells, resulting in the generation of ROS.

The electrolytic effects on CT-26 cells were further assessed by flow cytometry (Figure S8a) and real-time CLSM monitoring of cell apoptosis using Annexin V-FITC and PI. Cells were incubated with PBS (100 μL) and DFA IV-PEI–PEG-GNRs (50 μg/mL) for 12 h, exposed to a square-wave DC field for multiple time intervals, and double-stained with Annexin V-FITC/PI. The DFA IV-PEI–PEG-GNRs + EF (30 min)-treated cells showed a 90.28% apoptosis rate, which was higher than that of the control (2.59%), control + EF (30 min, 28.65%), and DFA IV-PEI–PEG-GNRs (14.13%), suggesting a significant electrolytic effect of the DFA IV-PEI–PEG-GNRs under an electric field (Figure S8b). To further identify cell apoptosis, Figure 5a shows Annexin V-FITC/PI double-staining fluorescence images and the fluorescence intensity variation of PBS-and DFA IV-PEI–PEG-GNR-treated cells around the cathode and anode after application of the DC field at various time intervals. After applying a DC electric field for 13 min, DFA IV-PEI–PEG-GNR-treated cells at the cathode exhibited cell swelling and membrane rupture due to electroosmotic dehydration compared with the control (22 min, Video S17), which showed a strong red fluorescence because most of the cells had died, implying that rapid cell death at 13 min (Video S18) occurred because the DFA IV-PEI–PEG-GNRs served as electric nanoantennas under a DC field. Figure 5b shows PI fluorescence from DFA IV-PEI–PEG-GNR-treated cells at the cathode, which was approximately 10 min faster than that of the controls. Near the anode, DFA IV-PEI–PEG-GNR-treated cells showed significant red fluorescence after 18 min (Video S19) compared with the control (Video S20), indicating their potential electrolytic cancer ablation ability. The PI fluorescence intensity of DFA IV-PEI–PEG-GNR-treated cells at the anode was much higher than that of the control (Figure 5c).

Caspase 3/7 (green) and tetramethylrhodamine methyl ester (TMRM) (red) staining were used to confirm cell apoptosis. Caspase 3/7 is a fluorescent dye that binds to nucleic acids. Bright green fluorescence indicated apoptotic cells with activated caspase 3/7, whereas cells without activated caspase 3/7 showed low fluorescence. TMRM is a negatively charged red fluorescent dye that labels healthy, undamaged mitochondria and is released from cancer cells when their mitochondrial membrane potential is damaged.^37^Figure S9 shows caspase 3/7 and TMRM double-staining fluorescence micrographs of PBS (100 μL)-and DFA IV-PEI–PEG-GNR (50 μg/mL)-treated cells treated with EA. At the cathode and anode, DFA IV-PEI–PEG-GNR-treated cells exhibited bright green fluorescence compared to control cells under the same treatment conditions, indicating that caspase 3/7 was likely to cause apoptosis. The control cells showed strong red TMRM signals in healthy mitochondria at the cathode and anode. In contrast, DFA IV-PEI–PEG-GNR-treated cells exhibited a decreased TMRM signal, indicating mitochondrial membrane potential damage.^38^

### DC Field Influences the Transformation of Nanoantennas and Inhibits Cell Growth

Toxicological studies of NPs are in high demand, with the first step being in vitro testing.^39^ Such studies have examined the interactions of NPs with cells cultured in DMEM, commonly supplemented with nutrients and antibiotics. NPs are not inert within this highly complex biological medium; they undergo various transformations, including adsorption of biomolecules, aggregation, and ion release.^40^ These mechanisms are required for various biological reactions at the cellular level. To address these phenomena, an initial approach is the characterization of NPs in terms of size to monitor transformations within the cell culture media. This characterization is intricate because of the kinetics of these processes and the complexity of DMEM. These transformations and interactions are undoubtedly influenced by differences in amino acid, glucose, and salt content.^40^ With this in mind, we dispersed DFA IV-PEI–PEG-GNRs (50 μg/mL) in DMEM as described.^41^ The control (DMEM only) and DFA IV-PEI–PEG-GNRs dispersed in DMEM were treated with or without a square-wave DC field for 60 min at 37 °C. The DFA IV-PEI–PEG-GNRs (GNRs) were pelleted and assayed for the attachment of sulfur-containing biomolecules, such as cysteine (Cys), methionine (Met), tyrosine (Tyr), and phenylalanine (Phe), using an automatic amino acid analyzer attached to a Hitachi high-performance liquid chromatography (HPLC) system. This method provides information about size changes, biomolecule absorption, ion release, and dissolution, which are essential for assessing NP toxicity. Figure 5d shows the chromatograms of the amino acid profiles. Cys and Met concentrations were significantly decreased to 26% compared to the control (68%), indicating increased binding affinity mediated by surface sulfur-containing amino acids. These results suggest that this will also occur in EA and may play a major role in its efficacy. As previously reported, transformations of sulfur-containing amino acids contribute to metal transport, antioxidant activity, protein synthesis, DNA methylation, and gene expression.^42^ Met is an essential amino acid that improves metabolism, prolongs life span, and prevents and suppresses cancers.^43^

TEM was used to assess the size and aggregation of DFA IV-PEI–PEG-GNRs. DFA IV-PEI–PEG-GNRs were dispersed in DMEM, with or without a square-wave DC field, for 60 min at 37 °C (Figure 5e). Those dispersed in DMEM showed more surrounding organic matter after the DC field, which eventually formed a core–shell type structure, consistent with sulfur-containing biomolecules that enhance the binding of other materials. In contrast, those that were not subjected to the DC field showed no significant changes.^44^ CT-26 cells were incubated with or without DFA IV-PEI–PEG-GNRs (50 μg/mL) under a square-wave DC field for 20 min and then incubated for 24, 48, and 72 h. The cells were costained with calcein-AM and PI for 30 min and visualized using CLSM. As shown in Figure 5f, DFA IV-PEI–PEG-GNR treatment inhibited growth under the DC field, while the control lacking GNRs exhibited significant growth. Figure 5g illustrates a model for the effects of EA on CT-26 cells, leading to the generation of reactive oxygen species (ROS).

### Impact of Nanoantenna Delivery on Colorectal Cancer Cell Phenotype

The effects of EA using DFA IV-PEI–PEG-GNRs on the cellular morphology and their interactions with organelles were examined using bio-TEM. Untreated control CT-26 cells displayed normal nuclei, mitochondria, Golgi apparatus, heterochromatin, and microvilli, with intact nuclear membranes, regular shapes, and smooth surfaces (Figure S10a). Bio-TEM images of CT-26 cells treated with DFA IV-PEI–PEG-GNRs (highlighted with red arrows) without DC are presented in Figure S10b,c. These particles entered the cells and accumulated inside organelles, while the cells maintained normal mitochondria, and some organelles showed regular shapes and smooth surfaces, indicating low toxicity. In contrast, under a DC field, DFA IV-PEI–PEG-GNRs induced morphological changes at the cathode, including injury, inactivation, irregular cell membranes, membrane blebbing on the cell surface, reduction in microvilli, cytoplasmic condensation, nuclear fragmentation, and fragmented mitochondria (green arrowheads), ultimately leading to cell death. The loss of microvilli and the presence of cellular debris containing apoptotic bodies (AB) are depicted in Figure S11a (marked by a black arrow). Double-membraned autophagosomes (single white arrow) and single-membraned autolysosomes (double white arrows) are present in the ER; (blue arrows) due to stress induced by the DC field (enclosed in a red box). ER stress (enclosed in a yellow box) and damage-induced fusion of inner membranes and cristae structures (yellow arrowhead) in mitochondria (yellow arrow) are also noticeable. These changes were evident in the presence of vacuolated and swollen organelles containing the transfected GNRs (red arrows). The electron-dense organelles appeared to be healthy mitochondria (M) as they displayed a double membrane compartment (black arrowheads). The blue box shows vacuolated and swollen mitochondria with mitochondria-derived vesicles (orange arrow) at the outer membrane and damaged cristae. Figure S11b,c reveal morphological alterations observed midway between the electrodes, similar to those near the cathode. Between the electrodes (red box), we observed swollen and fragmented Golgi (G) apparatus (purple arrow), secretory vesicle release, web-like microtubules (marked by an asterisk), and lysed mitochondria. As shown in Figure S11d–f, more severe damage was observed in small nuclear fragments (N*), mitochondrial inner membrane, and cristae near transfected GNRs at the anode, indicating that the uptake of DFA IV-PEI–PEG-GNRs was facilitated by conjugation.

### Intracellular ER Stress-Mediated Calcium Accumulation and ROS Generation

To evaluate the intracellular calcium (Ca^2+^) and ROS productivity of DFA IV-PEI–PEG-GNRs under a DC field, we used Fluo-4 AM (green) and CellROX Deep Red staining. Fluo-4 AM, a high-affinity Ca^2+^ fluorescent dye, can detect and quantify cellular Ca^2+^ changes in both the whole cell and subcellular regions.^45^ Ca^2+^ is released from the ER because of ER stress, which subsequently enters the mitochondria and induces mitochondrial ROS generation. To evaluate intracellular ROS generation and oxidative stress, we used CellROX Deep Red, a fluorogenic probe (nonfluorescent while in a reduced state and bright fluorescence upon oxidation by ROS).^46^ After treatment with DFA IV-PEI–PEG-GNRs (50 μg/mL) for 12 h, the cells were stained for 30 min at 37 °C and subjected to a square-wave DC field multiple times. As shown in Figure S12a–d, time-dependent changes were observed at both the cathode (Figure S13a) and anode (Figure S13b). Specifically, there was strong green fluorescence at the cathode (Videos S21 and S22) and anode (Videos S23 and S24) compared to the control cells at the cathode (Videos S25 and S26) and anode (Videos S27 and S28). These observations indicate ER stress-mediated Ca^2+^ accumulation in the mitochondria and ROS generation. In the presence of ROS, DFA IV-PEI–PEG-GNR-treated CT-26 cells were quickly oxidized and displayed strong red fluorescence at the cathode (Videos S29 and S30) and anode (Videos S31 and S32), compared to the control at the cathode (Videos S33 and S34) and anode (Videos S35 and S36). This indicated efficient ROS production.

### In Vivo Electrolytic Tumor Ablation Therapy

**Figure 6:**
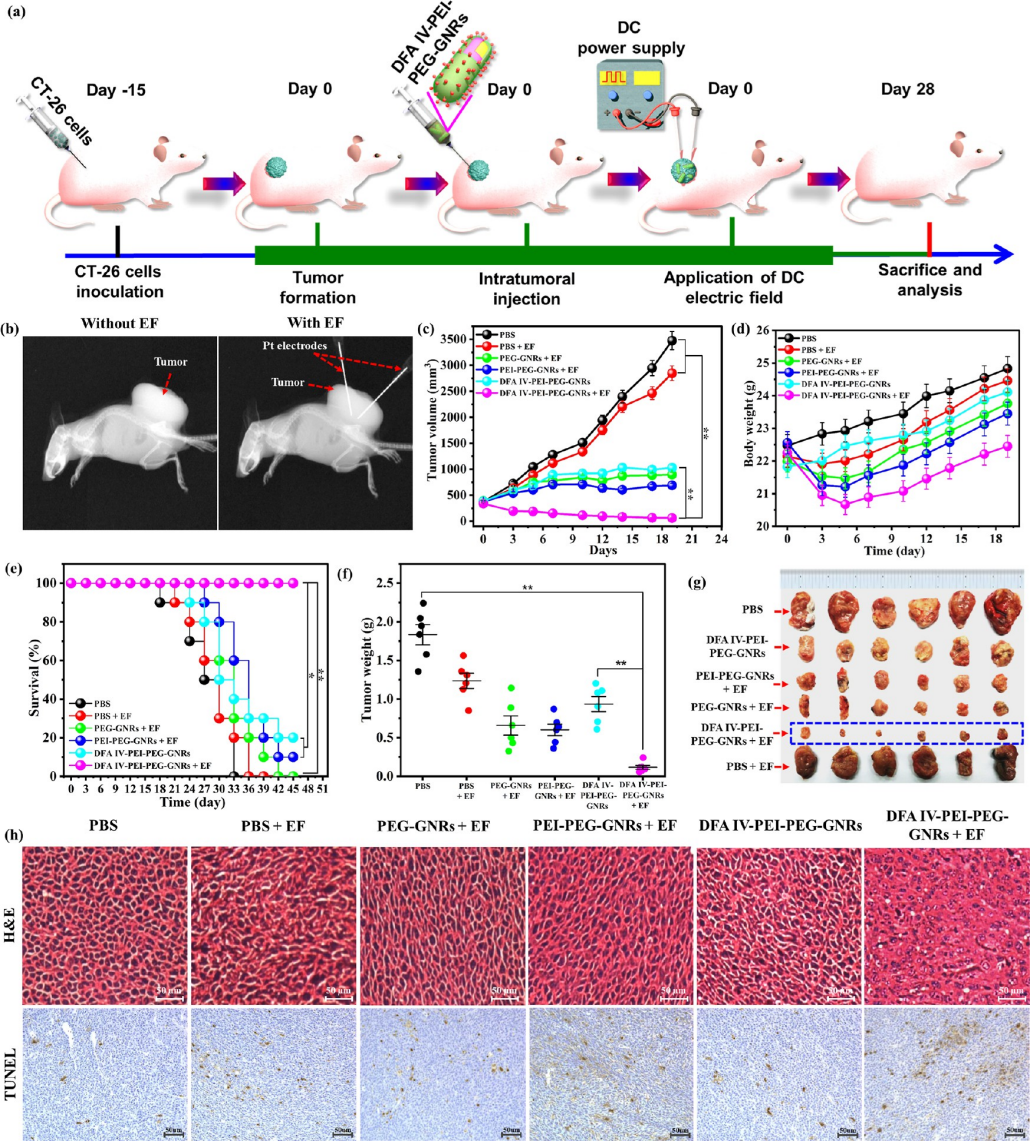
In vivo electrolytic tumor ablation therapy. (a) Demonstration of electrolytic tumor ablation therapy protocol in mice. (b) X-ray image of CT-26 tumor-bearing mice after intratumoral injection of DFA IV-PEI–PEG-GNRs, with or without a square-wave DC field for 30 min. (c) Average tumor volume in mice after various treatments. *p* values: **p < 0.01. (d) Body weights of mice in various groups after treatment. (e) Survival curves of CT-26 tumor-bearing mice after the different treatments. *p* values: *p < 0.05, **p < 0.01. Average tumor weight (f) and photographs (g) of tumors harvested on the 28th day from various treatment groups. *p* values: **p < 0.01. (h) H&E and TUNEL staining of tumor tissues harvested on the 28th day from various treatment groups of mice.

EA using DFA IV-PEI–PEG-GNRs was assessed in CT-26 tumor-bearing nude mice, according to the protocol outlined in Figure 6a. Three weeks after the subcutaneous injection of cells, the tumors reached approximately 300 mm^3^. Tumor-bearing mice were then randomly assigned to treatment groups, with six animals per group: (1) control (100 μL PBS only), (2) control (100 μL PBS) + EF, (3) PEG-GNRs (50 μg/mL) + EF, (4) PEI–PEG-GNRs (50 μg/mL) + EF, (5) DFA IV PEI–PEG-GNRs (50 μg/mL) only, and (6) DFA IV PEI–PEG-GNRs (50 μg/mL) + EF. Following intratumoral injection, a square-wave DC field was generated at 1 mA (4.3 V/cm) using two Pt electrodes inserted 1.0 to 3.0 cm in the tumor region for 30 min and imaged by X-ray, as depicted in Figure 6b. Tumor pH was measured using a pH electrode with a pH meter connected to two Pt electrodes to evaluate the changes in tumor pH in response to EA using DFA IV PEI–PEG-GNRs (50 μg/mL), which created drastic pH variations in the anode and cathode, resulting in cell death (Figure S14). The tumor volume (Figure 6c), body weight (Figure 6d), and survivability rates (Figure 6e) were measured. Tumor volume significantly differed between the groups. EA with DFA IV PEI–PEG-GNRs suppressed tumor growth, whereas DC treatment with PBS, PEG-GNRs, PEI–PEG-GNRs, PBS alone, or DFA IV PEI–PEG-GNRs alone did not significantly inhibit tumor growth. Compared with previously reported cancer treatment approaches based on EA, EA using DFA IV PEI–PEG-GNRs exhibited a significant reduction in tumor growth (Table S1). The six groups showed no apparent differences in body weight and no mice died during the experiment. Survival was assessed over 45 days, showing that was significantly enhanced by EA using DFA IV PEI–PEG-GNRs, whereas the other groups exhibited significantly lower survival rates. On day 28, all mice were euthanized, and the tumors were excised, measured, weighed, and photographed (Figure 6f,g). Both tumor weight and volume were substantially lower in mice treated with DFA IV PEI–PEG-GNRs and DC fields than in other groups. Our tumor weight and volume were similar to a previous report using doxorubicin-loaded PEG-coated porous Pt NPs + square wave electric field, which was much lower than that in other groups.^47^

To further verify the efficacy of DFA IV PEI–PEG-GNRs, hematoxylin and eosin (H&E)- and TUNEL-stained tumor sections were histologically examined. Tumor tissues from mice treated with DFA IV PEI–PEG-GNRs combined with square-wave DC treatment exhibited significantly more damage than those from other groups (Figure 6h), confirming the efficacy of DFA IV PEI–PEG-GNRs.^10^ The TUNEL assay clearly showed more apoptotic cells in the DFA IV PEI–PEG-GNRs with square-wave DC treatment than in the other groups, indicating that DFA IV PEI–PEG-GNRs have great potential as electrolytic materials for cancer therapy (Figure S15). Furthermore, the histopathological results of the major organs (heart, kidney, liver, lung, and spleen) were analyzed using H&E staining (Figure S16). All histopathological images showed well-defined cytoplasm and nuclei in the DFA IV PEI–PEG-GNRs + EF group, which had no obvious histopathological abnormalities compared with the PBS + EF group, indicating that DFA IV PEI–PEG-GNRs showed no noticeable toxic effects on the major organs of mice and offers a potential approach with the advantages of excellent biocompatibility and biosafety for highly efficient electrolytic tumor ablation therapy of CT-26 cells. To evaluate biosafety, fresh blood was collected to examine white blood cell count, red blood cell count, hematocrit, hemoglobin, mean platelet volume, mean corpuscular hemoglobin, mean corpuscular hemoglobin concentration, and platelet count (Figure S17). All tests yielded results similar to those of the control groups, demonstrating that DFA IV PEI–PEG-GNRs are an effective and safe electrolytic material for cancer therapy.^48^

To further measure the biodistribution of nanomaterials in vivo, the amount of Au in the blood, tumors, and major organs (heart, lung, spleen, kidney, and liver) 24 h postintratumoral injection was determined using AAS (Figure S18). Significantly, the Au concentrations of DFA IV PEI–PEG-GNRs + EF were quantified to be 9.03 ± 0.45 ID/g, which was much higher than that of other treatment groups. Meanwhile, high Au concentrations were observed in the spleen (4.43 ± 0.22 ID/g) and liver (3.55 ± 0.17 ID/g), which were reticuloendothelial systems responsible for the metabolism, degradation, and clearance of nanomaterials, indicating that DFA IV PEI–PEG-GNRs were efficiently and safely cleared from the body.

## Conclusions

In this study, we developed a class of electric nanoantennas and advanced nanoelectrocatalysts for Electrochemical Ablation (EA), known as DFA IV-PEI–PEG-GNRs. Our aim was to leverage the promising capabilities of GNRs with enhanced biological compatibility and improved targeting capabilities; thus, we introduced DFA IV-PEI–PEG modification. The DFA IV-PEI–PEG-GNRs possess several noteworthy attributes that make them highly efficient in the context of EA. They display superior catalytic ability, enabling them to accelerate chemical reactions without being consumed, which enhances the rate of the electrolysis process. This is crucial in EA as it facilitates the generation of key reaction products that destroy tumor cells.

In addition, the modified GNRs exhibited excellent conductivity, which is an essential feature of EA. This trait allows them to effectively conduct the electrical current necessary for electrochemical reactions that occur during EA. Versatility is another important characteristic that indicates their capability to perform under various conditions and circumstances, potentially broadening the scope of their applications. They also demonstrated impressive biocompatibility and biosafety, ensuring that they do not induce adverse effects in the biological system to which they are introduced. Moreover, they have demonstrated strong near-infrared (NIR) absorption capability, a highly desirable property for nanomaterials used in cancer treatment, because of their potential for targeted hyperthermia treatments. Their stability ensures functionality and structural integrity under treatment conditions.

One of the most exciting attributes of DFA IV-PEI–PEG-GNRs is their high tumor-targeting ability. This characteristic allows these nanoantennas to localize at the site of the tumor, improving the effectiveness and reducing potential damage to the surrounding healthy tissue. When a DC was applied, DFA IV-PEI–PEG-GNRs accelerated the dissociation of water molecules and produced a host of electrochemical reaction products such as hydrogen (H_2_), protons (H^+^), sodium hydroxide (NaOH), chlorine gas (Cl_2_), hypochlorous acid (HClO), hydrochloric acid (HCl), and oxygen (O_2_). These reaction products cause significant changes in the pH environment of tumor tissues, leading to an extremely high nano electrocatalytic efficiency that is lethal to tumor cells.

Our results indicate that these modified GNRs show significant efficacy against both cultured cancer cells and large solid tumors after DC treatment, offering a promising route for efficiently treating large-scale tumors. Hence, we foresee that DFA IV-PEI–PEG-GNRs will be a promising and effective nanoparticle for EA treatment in patients with relatively large initial tumors. We conclude that our study could advance the clinical development of EA, offering a potent and targeted method for cancer treatment.

## Methods

### Chemical and Reagents

Gold(III) chloride trihydrate (HAuCl_4_·3H_2_O, 99.9%), sodium borohydride (NaBH_4_), hexadecyltrimethylammonium bromide (CTAB, 99%), benzyldimethylhexadecylammonium chloride, silver nitrate (AgNO_3_), ammonium hydroxide solution (NH_4_OH, 30–33%), l-ascorbic acid, *N*-hydroxysuccinimide (NHS), thiol-PEG-carboxyl (SH-PEG3500-COOH, *M*_n_ = 3500), branched PEI (*M*_w_ ∼ 800), 1-ethyl-3-(3-(dimethylamino)propyl) carbodiimide hydrochloride (EDC), and MTT were purchased from Sigma-Aldrich (St. Louis MO, USA). Di-β-D-fructofuranose-2,6’:6,2′-dianhydride (DFA IV) was purchased from Real Biotech Co. Ltd. (Chungnam, South Korea). DMEM, penicillin–streptomycin (10,000 units/mL of penicillin and 10,000 μg/mL of streptomycin), heat-inactivated FBS, and trypsin–ethylenediaminetetraacetic acid (EDTA) solution (0.05% trypsin and 0.53 mM EDTA·4Na) were purchased from Welgene Inc. (Gyeongsangbuk-do, Korea). Ultrapure water (18.2 MΩ cm) was produced using an AquaMAX Ultra 370 series water purification system (Young Lin Instrument Co. Ltd., Gyeonggi-do, Korea) and used for all experiments. All other biological reagents were supplied by Thermo Fisher Scientific, Inc. (Waltham, MA, USA). All chemicals were of analytical grade and were not further purified before use. Chamlide EC perfusion-type electric stimulation magnetic chamber was obtained from Live Cell Instruments (Byeollae-dong, Korea).

### Synthesis of DFA IV-PEI–PEG-GNRs

Seed-mediated growth using CTAB as the capping agent was used to synthesize CTAB-capped GNRs as described by Murphy and El-Sayed.^23,49^ After synthesis, the GNR solution was centrifuged at 11,000*g* for 25 min and washed three times to remove excess CTAB. Thiol-PEG-carboxyl (SH-PEG-COOH) was conjugated to the GNR core surface to produce PEG-modified GNRs (PEG-GNRs) as previously described.^24^ The purified GNRs (20 mL) were modified with thiol-PEG linkers (SH-PEG-COOH, 2 mg/mL) via gold–thiol interactions and stirred overnight. PEG-GNR solution was centrifuged at 11,000*g* for 25 min to remove excess PEG and was stored at 4 °C until further use. For the amine functionalization of the PEG-GNR surface, the primary amine groups of PEI (0.4 mL) were reacted with carboxyl-PEG-GNRs (20 mL) using 1.0 mg of EDC/NHS (1:1) and stirred for 24 h at room temperature (RT, 25 °C). PEI–PEG-GNRs were centrifuged, washed three times with DW, and redispersed in DW for further use. DFA IV (2 mL, 10 mg/mL) was conjugated to PEI–PEG-GNRs (20 mL) under continuous stirring for 24 h. The resulting DFA IV-PEI–PEG-GNRs were purified thrice by centrifugation at 11,000*g* for 25 min and then stored at 0 °C.

### Characterization

The UV–vis absorption spectra of the GNRs, PEG-GNRs, PEI–PEG-GNRs, and DFA IV-PEI–PEG-GNRs were measured using a UV-2600 spectrometer (Shimadzu Ltd., Tokyo, Japan). The morphologies of GNRs, PEG-GNRs, PEI–PEG-GNRs, and DFA IV-PEI–PEG-GNRs were investigated using transmission electron microscopy (TEM; JEM 2100, JEOL, Tokyo, Japan). Scanning TEM (STEM), field emission TEM (FETEM), and energy dispersive X-ray spectroscopy (EDX) and mapping were performed using a Cs-corrected JEOL JEM-ARM200F microscope operated in both the FETEM and STEM modes at 80 kV. The particle size distribution and surface charge of all the samples were determined using dynamic light scattering (DLS, Brookhaven 90 Plus particle sizer, Brookhaven Instruments, NY, USA) and a Brookhaven ZetaPALS ζ-potential analyzer. Fourier-transform infrared (FTIR) spectra of the samples were recorded using a Thermo Fisher Scientific Nicolet iS50 FTIR spectrometer. X-ray diffraction (XRD) patterns of the samples were obtained using a Rigaku (Miniflex, Japan) X-ray diffractometer with Cu–Kα radiation. The gold (Au) concentrations in the samples were measured by AAS (MegaA-700FG, Scinco, Gangnam-gu, Korea). Tumor-bearing mice were imaged using X-rays (MAMMO X-ray Unit, 100 kHz). The stability tests of PEG-GNRs, PEI–PEG-GNRs, and DFA IV-PEI–PEG-GNRs were performed in DW, PBS, and DMEM without phenol red with 10% FBS, and the solutions were determined using a UV-2600 spectrometer at room temperature for 7 days.

### Electrolytic Ablation

The therapeutic effects of EA were evaluated using an experimental setup (Figure S19). Live cell imaging was performed using a live cell incubator system with a Pt electrode and suction needle (Chamlide EC, Live Cell Instrument, Korea) to maintain standard cell culture conditions (37 °C, 5% CO_2_) (Figure S20). A square wave DC power supply (PWS2323, Tektronix Inc., Korea) was used to set to a constant electric potential of 4.3 ± 0.1 V/cm and 1.0 milliamp (mA) at two Pt electrodes for various time intervals.

### High Biocompatibility and Effective Cellular Uptake of Gold Nanoantennas

The CT-26 murine colorectal carcinoma cell line was purchased from the Korean Cell Line Bank (Seoul, Korea) and maintained in Dulbecco’s modified DMEM medium supplemented with 10% FBS and 1% penicillin–streptomycin at 37 °C in a humidified 5% CO_2_ atmosphere. CT-26 cells were cultured in 48-well plates at a density of 4 × 10^5^/well (500 μL) and incubated overnight to allow for attachment. The cells were then incubated with multiple concentrations of GNRs, PEG-GNRs, PEI–PEG-GNRs, and DFA IV-PEI–PEG-GNRs (0–120 μg/mL) for 24 h. After treatment, cells were stained by adding MTT solution to insoluble formazan crystals, which were dissolved in dimethyl sulfoxide. The absorbance was measured at 570 nm using an Epoch microplate spectrophotometer (BIOTEK, Winooski, VT, USA). CT-26 cells (8 × 10^5^ cells/well) were plated in 35 mm dishes and cultured overnight. The cells were then incubated with DMEM containing PEG-GNRs (50 μg/mL), PEI–PEG-GNRs (50 μg/mL), and DFA IV-PEI–PEG-GNRs (50 μg/mL) for an additional 6, 12, 18, and 24 h. After incubation, the cells were harvested and digested with 1 mL of aqua regia, the and Au concentrations were quantified using AAS. We visualized the GNR distributions using dark-field inverted microscopy (Nikon Eclipse Ti–S, USA). Cells were plated in 35 mm culture dishes for 24 h to allow them to attach to the surface of the coverslips. The final concentrations of 50 μg/mL PEG-GNRs, PEI–PEG-GNRs, and DFA IV-PEI–PEG-GNRs were added to CT-26 cells and coincubated for 12 h. The cells were then washed with phosphate-buffered saline (PBS) and imaged under a dark-field inverted microscope.

### In Vitro Gas-Driven Electrolytic Ablation Therapy

CT-26 cells were seeded in 12-well plates at 6 × 10^5^/well, incubated overnight, and treated with PEG-GNRs, PEI–PEG-GNRs, or DFA IV-PEI–PEG-GNRs (0–50 μg/mL) for 12 h. The cells were connected to a square-wave constant DC potential of 4.3 V/cm and 1.0 mA for 30 min using two Pt electrodes. The cells were then provided with fresh DMEM and incubated for 24 h. A standard MTT assay was used to measure the cell viability. In the second assessment of ablation, CT-26 cells (6 × 10^5^) were plated on round coverslips in 35 mm culture dishes and cultured overnight. The cells were then incubated with PEG-GNRs, PEI–PEG-GNRs, and DFA IV-PEI–PEG-GNRs (50 μg/mL) for 12 h. The coverslips were transferred to Chamlide EC chambers and treated using a 4.3 V/cm electric potential and 1.0 mA using two Pt electrodes for 0, 10, 20, and 30 min. Cells were stained with calcein-AM and PI solution for 30 min to distinguish between live (green) and dead (red) cells using CLSM (Nikon A1 plus, Tokyo, Japan) in tail-scan mode. In the third assay, cells were seeded on round coverslips in 35 mm dishes, incubated overnight, and treated with PBS (100 μL) and DFA IV-PEI–PEG-GNRs (50 μg/mL) for 12 h. The coverslips were placed in Chamlide EC chambers and treated with a square-wave DC potential of 4.3 V/cm and 1.0 mA using two Pt electrodes for multiple time intervals. After treatments, cells were stained with Hoechst 33342 (27 μM, excited (Ex) at 361 nm), MitoTracker Green (1.0 μM, Ex at 490 nm), ER-Tracker Red (1.0 μM, Ex at 587 nm), and CellMask Deep Red Plasma membrane (×1, Ex at 649 nm) for 30 min to stain the cell nuclei, mitochondria, ER, and plasma membranes, respectively. The cells were rinsed three times with PBS and visualized using CLSM with a 60× oil-immersion objective. In the fourth assessment, CT-26 cells were cultured on glass coverslips in 35 mm dishes for 24 h. We prepared strongly acidic (pH 2.5 and 3.4) and alkaline (pH 10.3 and 11.4) DMEM; pH was adjusted with 1 M hydrochloric acid (HCl) or 1 M sodium hydroxide (NaOH). The cells were cultured in neutral DMEM with DFA IV-PEI–PEG-GNRs (50 μg/mL) for 12 h before altering the pH. The cells were stained with the four aforementioned strains. In the fifth assessment, cells were seeded on glass coverslips in 35 mm dishes and cultured overnight to assess apoptosis. After adding PBS (100 μL) and DFA IV-PEI–PEG-GNRs (50 μg/mL) for 12 h, cells were treated using a square-wave DC potential of 4.3 V/cm and 1.0 mA using two Pt electrodes for multiple time intervals. The cells were collected, washed with PBS, and stained with Annexin V-FITC/PI for 15 min. The cell suspensions were analyzed using a FACSVerse flow cytometer (BD Biosciences). Subsequently, the cells were costained with Annexin V-FITC (25 μL, Ex at 494 nm) and PI (5 μM, Ex at 535 nm) for 15 min. Coverslips were transferred to Chamlide EC chambers and imaged using CLSM. Apoptosis was confirmed using caspase 3/7 (green) and TMRM (red) staining. Cells were incubated with PBS (100 μL) and DFA IV-PEI–PEG-GNRs (50 μg/mL) for 12 h, treated with (4.3 V/cm) DC for 20 min, and stained with caspase 3/7 (2 μM, Ex: 502 nm) and TMRM (5 μM, EX: 548 nm) for 30 min. The cells were imaged using CLSM.

### Direct Current Field Influences Nanoantennas Transformation and Inhibits Cell Growth

NPs were dispersed as previously reported, with modifications.^44^ DFA IV-PEI–PEG-GNRs (50 μg/mL) were dispersed in DMEM by stirring for 60 min. The suspension was subjected to a square-wave DC field (4.3 V/cm) for 60 min at 37 °C, whereas DMEM only was used as a control without DC treatment. The control (DMEM only) and DFA IV-PEI–PEG-GNRs were pelleted and attached to Cys, Met, Tyr, and Phe and then analyzed using an automatic amino acid analyzer (Hitachi L-8900, Tokyo, Japan) attached to a Hitachi HPLC instrument. Additionally, the DFA IV-PEI–PEG-GNRs (50 μg/mL) dispersed in DMEM were treated with or without a square-wave DC field (4.3 V/cm) for 0, 30, 40, and 60 min at 37 °C and then characterized by TEM. CT-26 cells were seeded onto coverslips in 35 mm plates and incubated overnight. The cells were treated with or without DFA IV-PEI–PEG-GNRs (50 μg/mL) in a square-wave DC field for 20 min and then incubated for 24, 48, and 72 h. The cells were costained with calcein-AM and PI for 30 min. Cells were imaged using CLSM.

### Impact of Nanoantennas Delivery on Colorectal Cancer Cells’ Phenotype

The effect of DFA IV-PEI–PEG-GNRs (50 μg/mL) on CT-26 cell morphology was observed using a bio-TEM (Hitachi-HT7700, Tokyo, Japan). After treatment with DFA IV-PEI–PEG-GNRs (50 μg/mL) under a square-wave DC field (4.3 V/cm, 10 min), cells were collected, washed three times with PBS, fixed in 2.5% glutaraldehyde for 24 h followed by 1% Osmic acid (OsO_4_) for 1 h, dehydrated with ethanol, and embedded in Poly/Bed resin. Ultrathin sections (60–70 nm) were stained with 3% uranyl acetate and lead citrate and imaged using a Bio-TEM.

### Intracellular ER Stress-Mediated Calcium Accumulation and ROS Generation

Fluo-4 AM (green) was used to measure intracellular Ca^2+^ concentration. After treatment with DFA IV-PEI–PEG-GNRs (50 μg/mL) for 12 h, cells on coverslips were stained with 1 μM Fluo-4 AM for 30 min at 37 °C and then washed three times with DMEM to remove excess dye. Coverslips were placed on the bottom plate of the Chamlide EC chamber and treated with a square-wave DC field (4.3 V/cm) for multiple intervals, and then imaged by CLSM using 488 nm excitation and 510 nm emission filters. Fluorescence intensities were recorded for each cell in the field and expressed as relative Fluo-4 AM fluorescence. CellROX Deep Red reacts with ROS to produce a red fluorescence. Cells were plated overnight in 35 mm plates on coverslips and treated with DFA IV-PEI–PEG-GNRs (50 μg/mL) for 12 h. The cells were stained with CellROX Deep Red (5 μM, Ex at 644 nm) for 30 min. The coverslips were transferred to a Chamlide EC chamber, subjected to a square-wave DC field (4.3 V/cm), and imaged using CLSM. The fluorescence intensity per cell is expressed in relative units.

### In Vivo Electrolytic Tumor Ablation Therapy

Male BALB/c nude mice (5 weeks, 20 g) were obtained from Daehan Biolink (Chungbuk, Korea). All animal experiments were performed in accordance with the guidelines of the Animal Ethics Committee for Animal Experiments and were approved by the Institutional Animal Care and Use Committee (IACUC) of Woojung Bio (Approval Number WJIACUC20141117-4-07). To establish the CT-26 tumor model, 2 × 10^6^ cells suspended in 100 μL PBS were subcutaneously injected into the right rear legs of all mice. Mice were subjected to tumor ablation when their tumors grew to approximately 300 mm^3^, as measured using digital calipers. Tumor-bearing mice were randomly divided into six groups (*n* = 6) and treated with (1) control (100 μL PBS), (2) control (100 μL PBS) + electric field (EF; 4.3 V/cm), (3) PEG-GNRs (50 μg/mL) + EF (4.3 V/cm), (4) PEI–PEG-GNRs (50 μg/mL) + EF (4.3 V/cm), (5) DFA IV PEI–PEG-GNRs (50 μg/mL) only, and (6) DFA IV PEI–PEG-GNRs (50 μg/mL) + EF (4.3 V/cm). PBS (100 μL), PEG-GNRs (50 μg/mL), PEI–PEG-GNRs (50 μg/mL), and DFA IV PEI–PEG-GNRs (50 μg/mL) were intratumorally injected. After 10 min, groups 2, 3, 4, and 6 tumor-bearing mice were treated with a square-wave DC potential of 4.3 V/cm and 1.0 mA using two Pt electrodes inserted 1.0–3.0 cm in the tumor region for 30 min. Simultaneously, a pH electrode with a pH meter was connected to two Pt electrodes to successfully measure tumor pH. From the start of therapy, tumor volumes, and body weights were measured every 3 days. Survival was plotted using Kaplan–Meier graphs. On day 28, all mice were euthanized and the tumors were harvested, photographed, and weighed. Tumors were sectioned, stained with H&E, and subjected to TUNEL assay to detect histocompatibility and apoptotic cells, and imaged using CLSM. The major organs (heart, kidney, liver, lung, and spleen) were stained with H&E and imaged using CLSM. Blood was collected after euthanasia and biochemical tests were performed using a Coulter LH780 hematology analyzer (Beckman Coulter Inc., Carlsbad, CA, USA). For the in vivo biodistribution study, tumor-bearing mice were intratumorally injected with the aforementioned treatment groups and euthanized at 24 h. Blood, tumors, and major organs were harvested and digested with 1 mL of aqua regia, and Au concentrations were quantified using AAS.

### Statistical Analysis

OriginPro 2021 software (OriginLab Corp., Northampton, MA, USA) was used for all the statistical analyses. Data are presented as the mean ± standard deviation (SD). Statistical analyses were performed using one-way analysis of variance followed by Tukey’s posthoc test (*p < 0.05; **p < 0.01).

## Supporting Information Available

Schiff base DFA IV, synthetic chemical route of DFA IV-PEI–PEG-GNRs, cellular uptake, dark-field images, merged confocal fluorescence images, bio-TEM images, fluorescence intensities, intratumor pH, H&E staining, blood biochemical test, biodistribution of Au, experimental setup, and Chamlide EC chamber (PDF)
Merged images of confocal fluorescence images and bright field images of CT-26 cells treated with PBS (control) and DFA IV-PEI-PEG-GNRs under square wave DC field at different time intervals is discussed in Videos S1–S10 (ZIP)
Merged images of confocal fluorescence images and bright field images of DFA IV-PEI-PEG-GNRs-treated CT-26 cells at anode and when it is exposed to different pH values of DMEM at different time intervals are discussed in Videos S11–S15 (ZIP)
Merged images of confocal fluorescence images and bright field images of DFA IV-PEI-PEG-GNRs-treated CT-26 cells when it exposed to DMEM (pH = 11.4) and the CT-26 cells treated with PBS (control) and DFA IV-PEI-PEG-GNRs at anode or cathode under square wave DC field at different time intervals are discussed in Videos S16–S20 (ZIP)
Merged images of confocal fluorescence images and bright field images of Ca^2+^ and ROS generated by DFA IV-PEI-PEG-GNRs or PBS (control) at anode or cathode under square wave DC field at different time intervals are discussed in Videos S21–S30 (ZIP)
Merged images of confocal fluorescence images of ROS generated by DFA IV-PEI-PEG-GNRs or PBS (control)-treated CT-26 cells at the cathode with square-wave DC field for various time intervals is discussed in Videos S31–S36 (ZIP)
