# Supplementary material for: Electric Field-Responsive Gold Nanoantennas for the Induction of a Locoregional Tumor pH Change Using Electrolytic Ablation Therapy
Source: ACS Nano. 2024 Jul 8;18(30):19581–96. doi: 10.1021/acsnano.4c03610 (PMC11295197; doi:10.1021/acsnano.4c03610)
Supplement: Supplementary file 1 — nn4c03610_si_001.pdf [file nn4c03610_si_001.pdf]

Supporting information

Electric field-responsive gold nanoantennas for the induction of a locoregional tumor pH change using electrolytic ablation therapy

Ara Joe,[†,⊥] Panchanathan Manivasagan,[†,⊥] Jong Kook Park,[‡] Hyo-Won Han,[†] Sun-Hwa Seo,[†] Thavasyappan Thambi,[¶] Vu Hoang Giang Phan,[§] Soon Ah Kang,[‡] João Conde,[*,‖] Eue-Soon Jang[*,†]

[†] Department of Applied Chemistry, Kumoh National Institute of Technology, Daehak-ro 61, Gumi, Gyeongbuk 39177, Republic of Korea.

[‡] Department of Convergence Technology, Graduate School of Venture, Hoseo University, Seoul 06724, Republic of Korea.

[¶] Graduate School of Biotechnology, College of Life Sciences, Kyung Hee University, Yongin-si, Gyeonggi-do 17104, Republic of Korea.

[§] Biomaterials and Nanotechnology Research Group, Faculty of Applied Sciences, Ton Duc Thang University, Ho Chi Minh City, District 7, Vietnam.

[‖] ToxOmics, NOVA Medical School, Faculdade de Ciências Médicas, NMS|FCM, Universidade NOVA de Lisboa, Lisboa 1169-056, Portugal.

[⊥] These authors contributed equally.

*** Corresponding authors:** joao.conde@nms.unl.pt and euesoon@kumoh.ac.kr

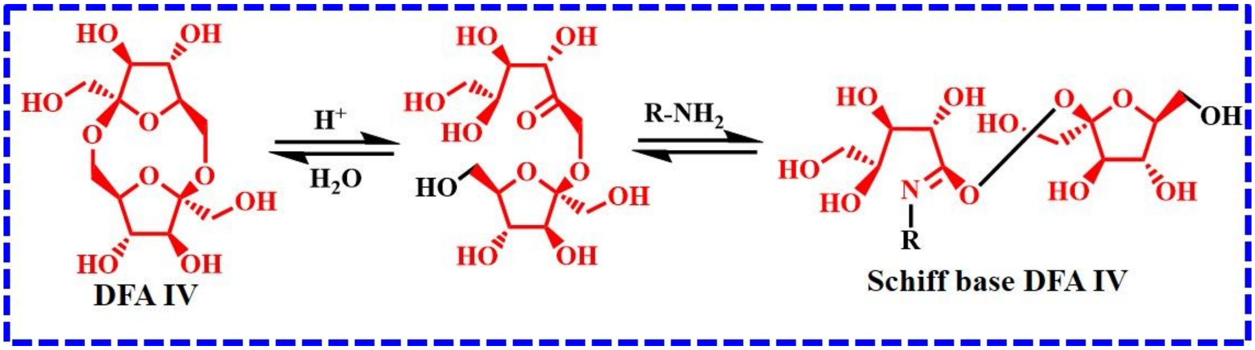

Figure S1. Synthetic route of Schiff base DFA IV.

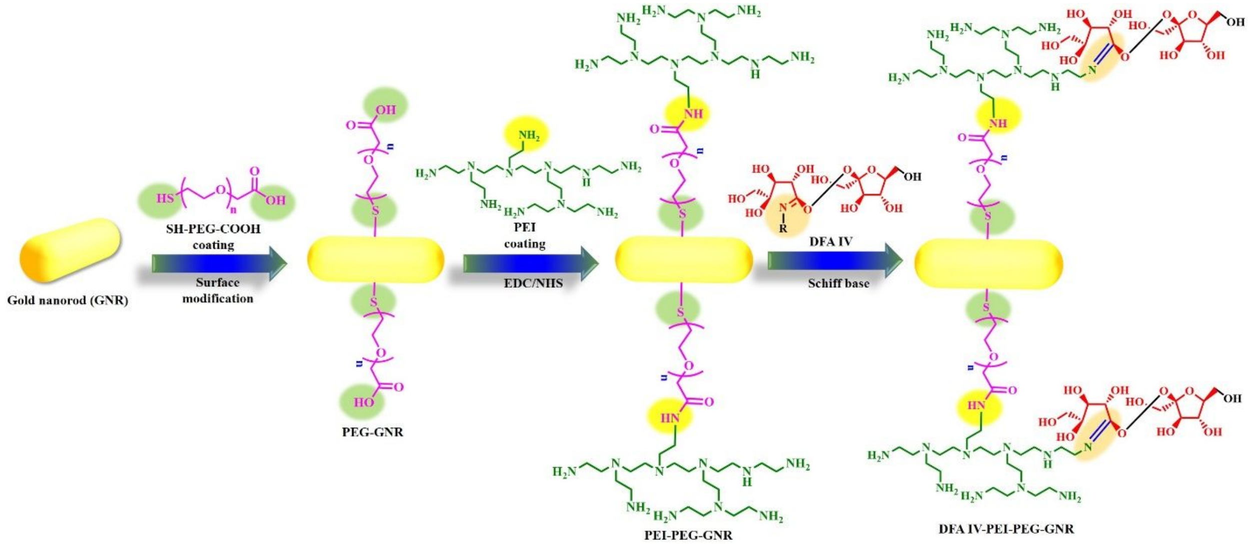

Figure S2. Synthetic chemical route of DFA IV-PEI-PEG-GNRs.

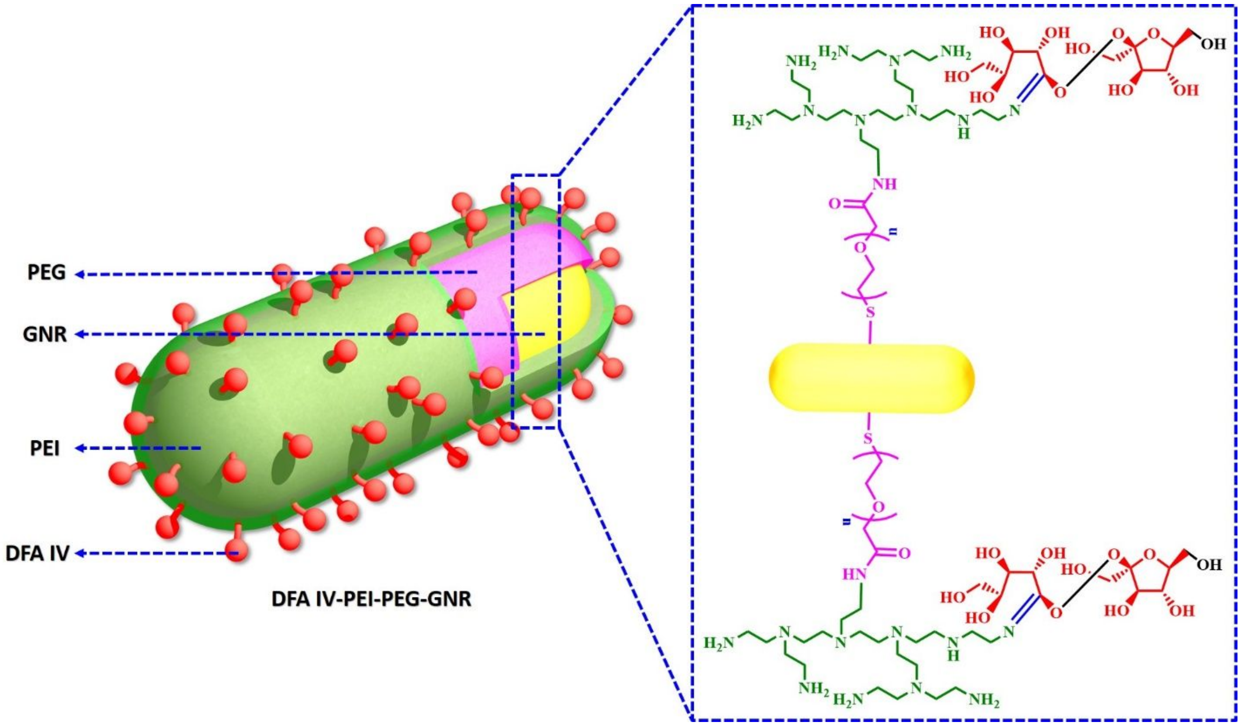

Figure S3. Schematic of the preparation of DFA IV-PEI-PEG-GNRs.

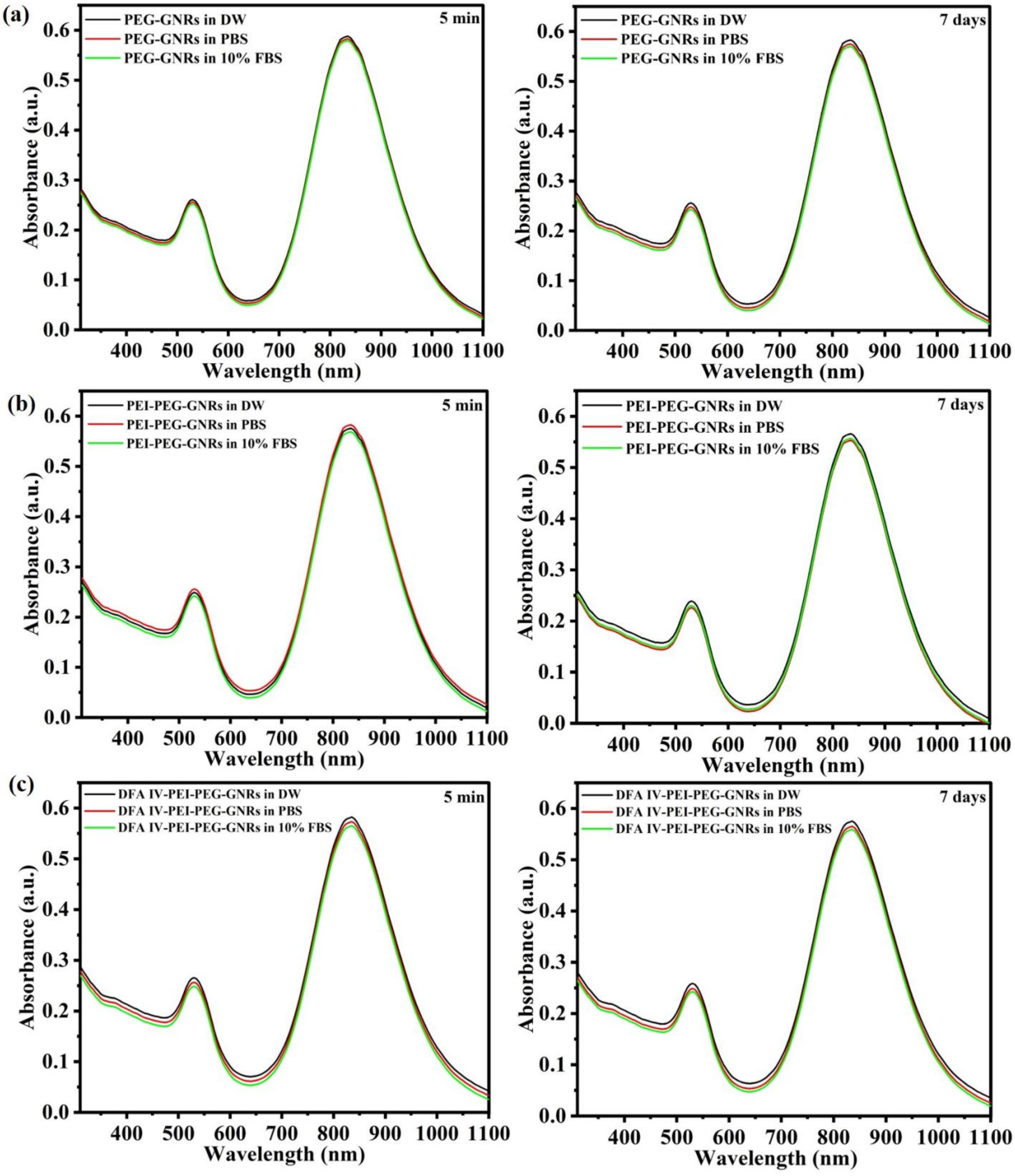

Figure S4. UV–vis–NIR spectra of PEG-GNRs, PEI-PEG-GNRs, and DFA IV-PEI-PEG-GNRs were dispersed in DW, PBS, and DMEM without phenol red with 10% FBS for 5 min and 7 days.

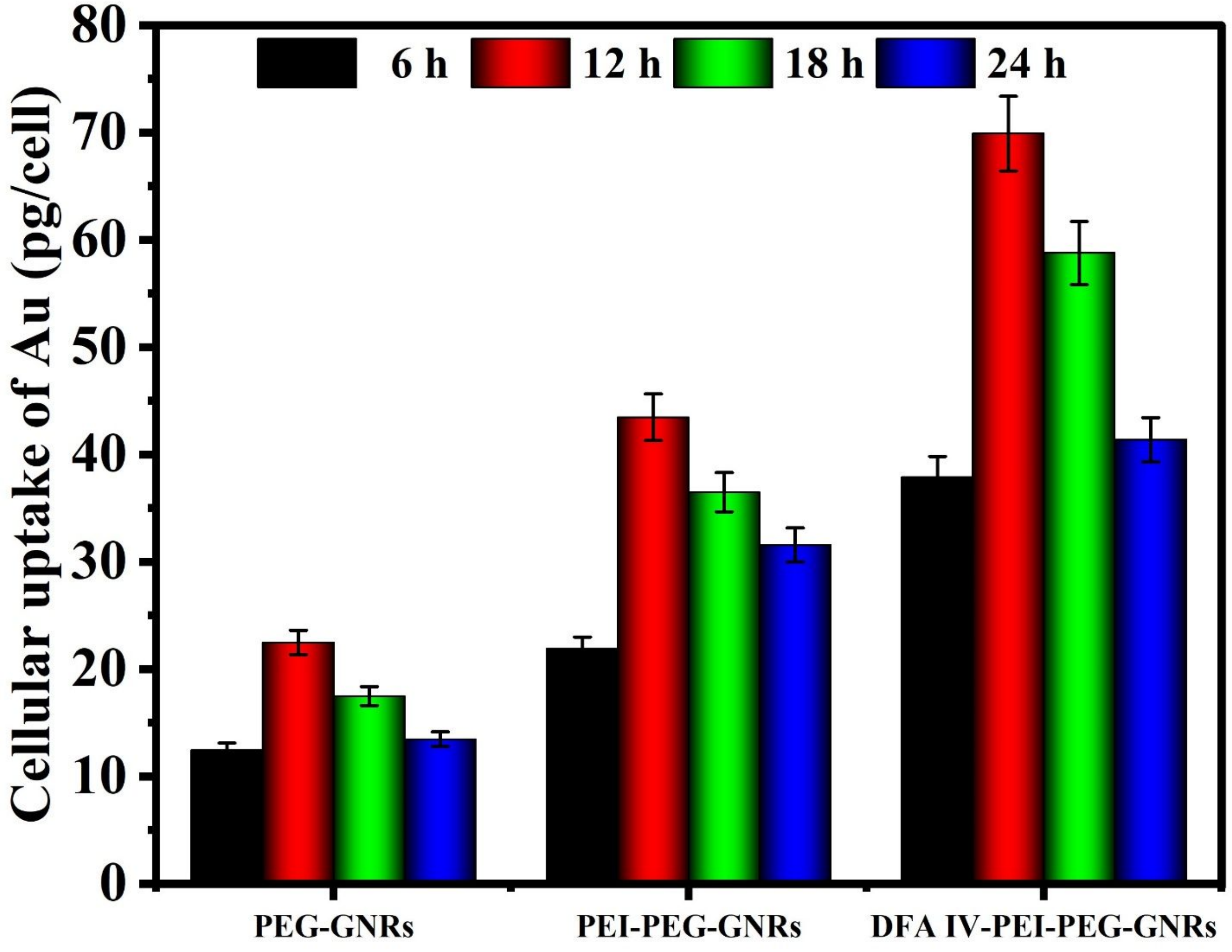

Figure S5. The cellular uptake of PEG-GNRs, PEI-PEG-GNRs, and DFA IV-PEI-PEG-GNRs in CT-26 cells was measured using AAS.

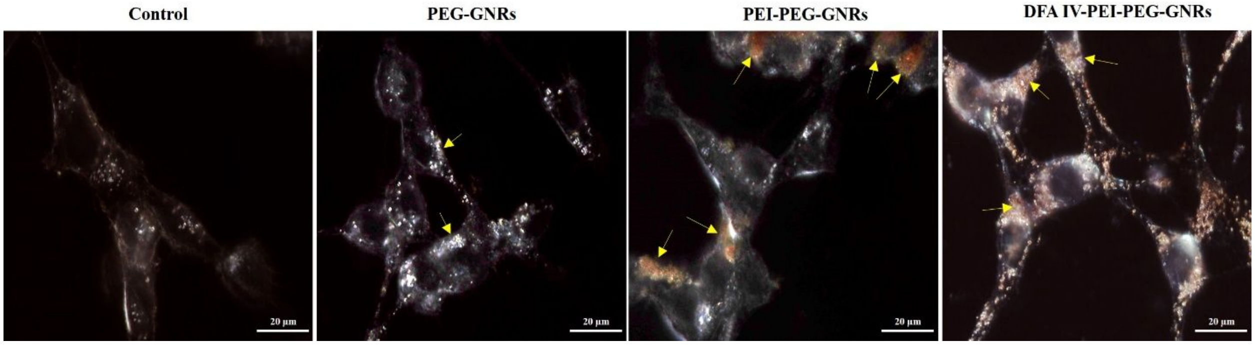

Figure S6. Dark-field images were used to visualize the distribution of PEG-GNRs, PEI-PEG-GNRs, and DFA IV-PEI-PEG-GNRs in CT-26 cells (60× magnification; scale bar: 20 μm). The yellow arrows indicate specific targeting of the NPs.

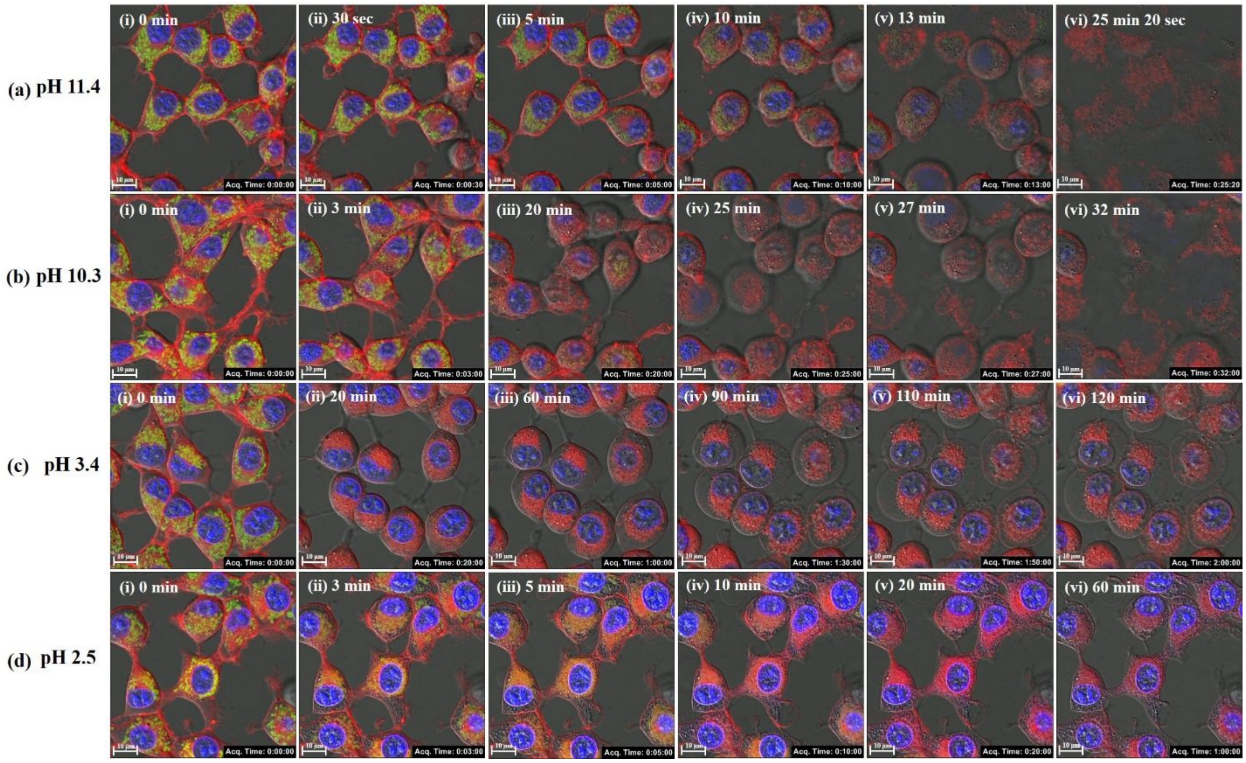

Figure S7. (a-d) Merged images of confocal fluorescence images and bright field images of DFA IV-PEI-PEG-GNR-treated cells exposed to strongly acidic (pH 2.5 and 3.4) and alkaline (pH 10.3 and 11.4) DMEM for multiple time intervals without a DC field (60× magnification; scale bar: 10 μm). Cells were stained with Hoechst 33342, MitoTracker Green, endoplasmic reticulum (ER)-Tracker Red, and CellMask Deep Red Plasma Membrane for 30 min to stain the nuclei, mitochondria, endoplasmic reticulum, and plasma membrane.

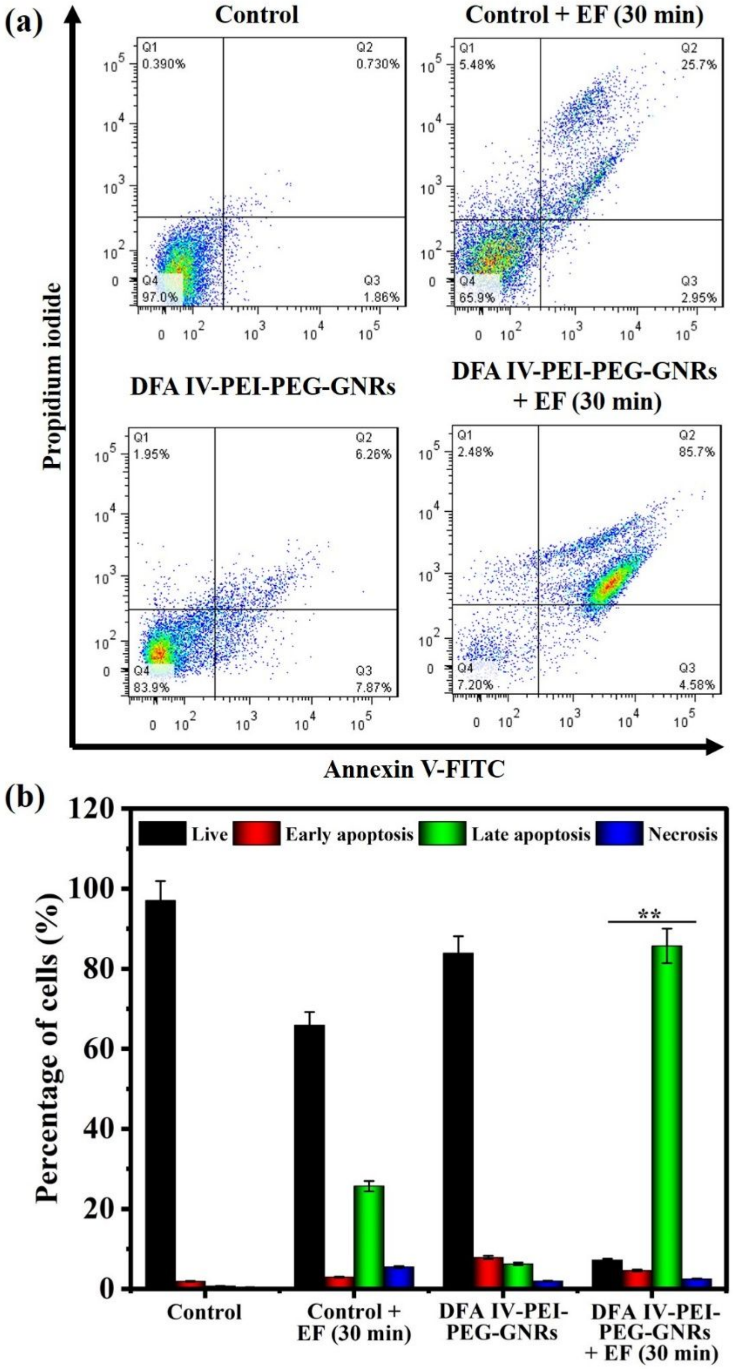

Figure S8. (a) Flow cytometry analysis of CT-26 cells apoptosis induced by control, control + EF (30 min), DFA IV-PEI-PEG-GNR alone, DFA IV-PEI-PEG-GNR + EF (30 min) using the Annexin-V/PI staining. (b) Apoptosis indexes are defined as apoptotic (early and late apoptotic) cells. *p* values: ** $p < 0.01$.

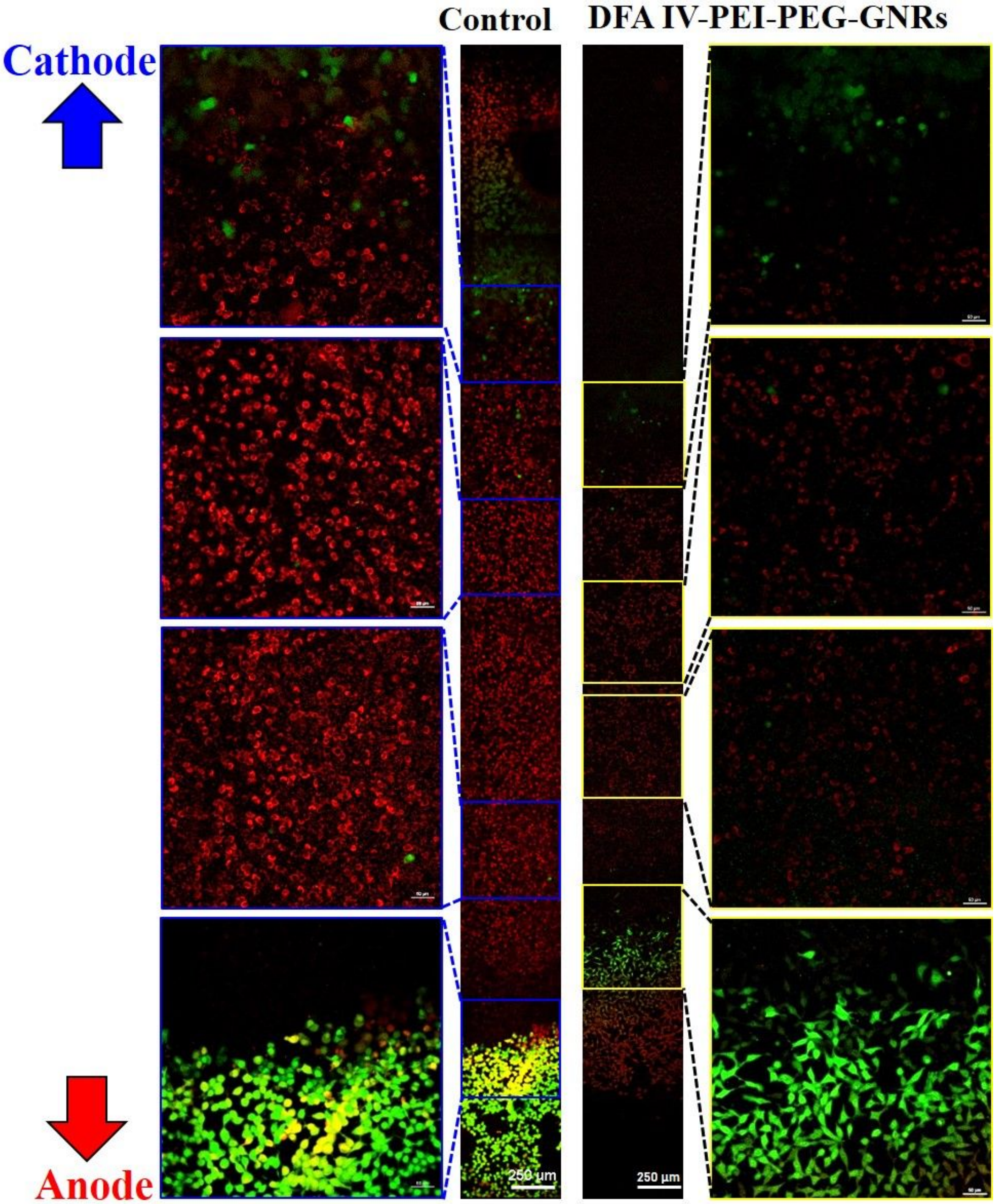

Figure S9. Merged confocal fluorescence images of CT-26 cell apoptosis and mitochondrial damage induced by PBS (control) and DFA IV-PEI-PEG-GNRs at the cathode and anode with a square-wave DC field using caspase 3/7 and TMRM double staining (20× magnification; scale bar: 250 μm).

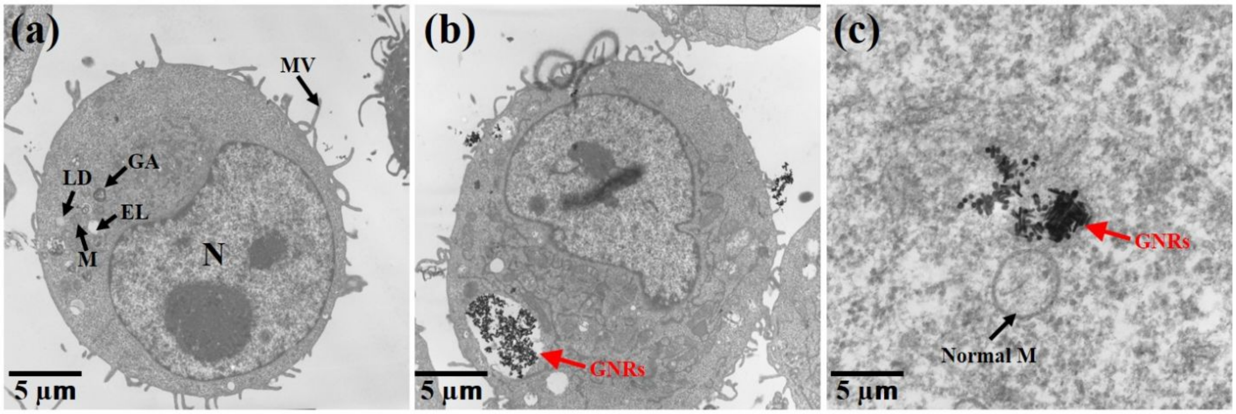

Figure S10. (a) Bio-TEM images of CT-26 cells treated with PBS alone (control). (b and c) Bio-TEM images of CT-26 cells treated with DFA IV-PEI-PEG-GNRs without DC. MV: microvilli of the cell surface, EL: electron-lucent vesicles, M: mitochondria, GA: Golgi apparatus, N: nucleus, D: lipid droplets, and GNRs: gold nanorods. Scale bars: 5 μm.

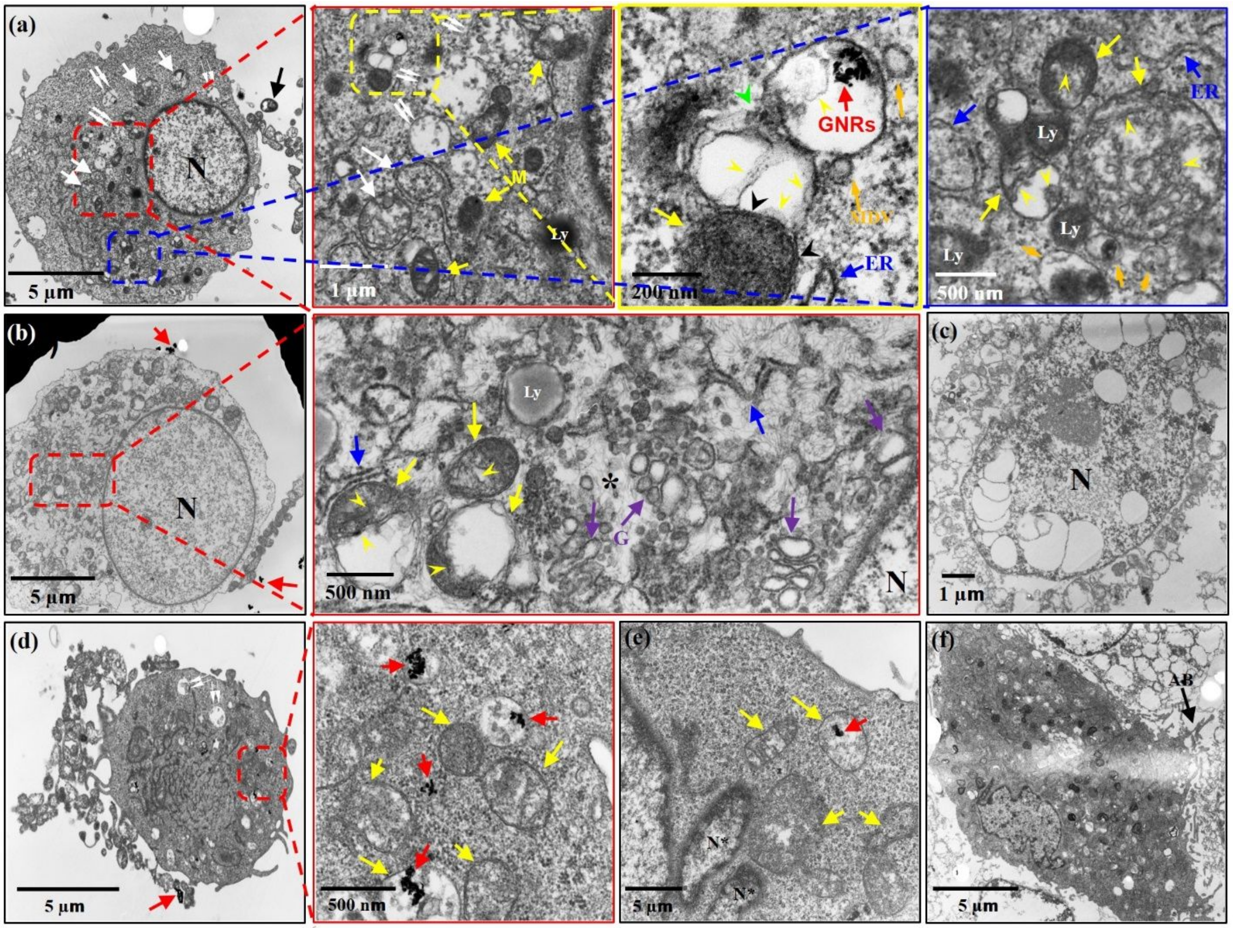

Figure S11. Bio-TEM images of CT-26 cells treated with DFA IV-PEI-PEG-GNRs at the cathode (a), middle (b,c), and anode (d-f) under DC treatment for 10 min. The images show several morphological changes. N: nucleus, M: mitochondria, Ly: lysosome, ER: endoplasmic reticulum, G: Golgi apparatus, *: microtubules, N*: fragments of the nucleus, MDV: mitochondria-derived vesicles, and AB: apoptotic bodies. The red arrows indicate the gold nanorods (GNRs); the green arrowhead indicates the fragmented mitochondria; the black arrow and black arrowheads indicate the apoptotic bodies and double-membrane compartment; the single and double white arrows indicate the double-membraned autophagosomes and single-membraned autolysosomes; the blue arrows indicate the endoplasmic reticulum; the yellow arrow and yellow arrowhead indicate the mitochondria and cristae; the orange arrow indicates the mitochondria-derived vesicles; and the purple arrow indicates the fragmented Golgi apparatus.

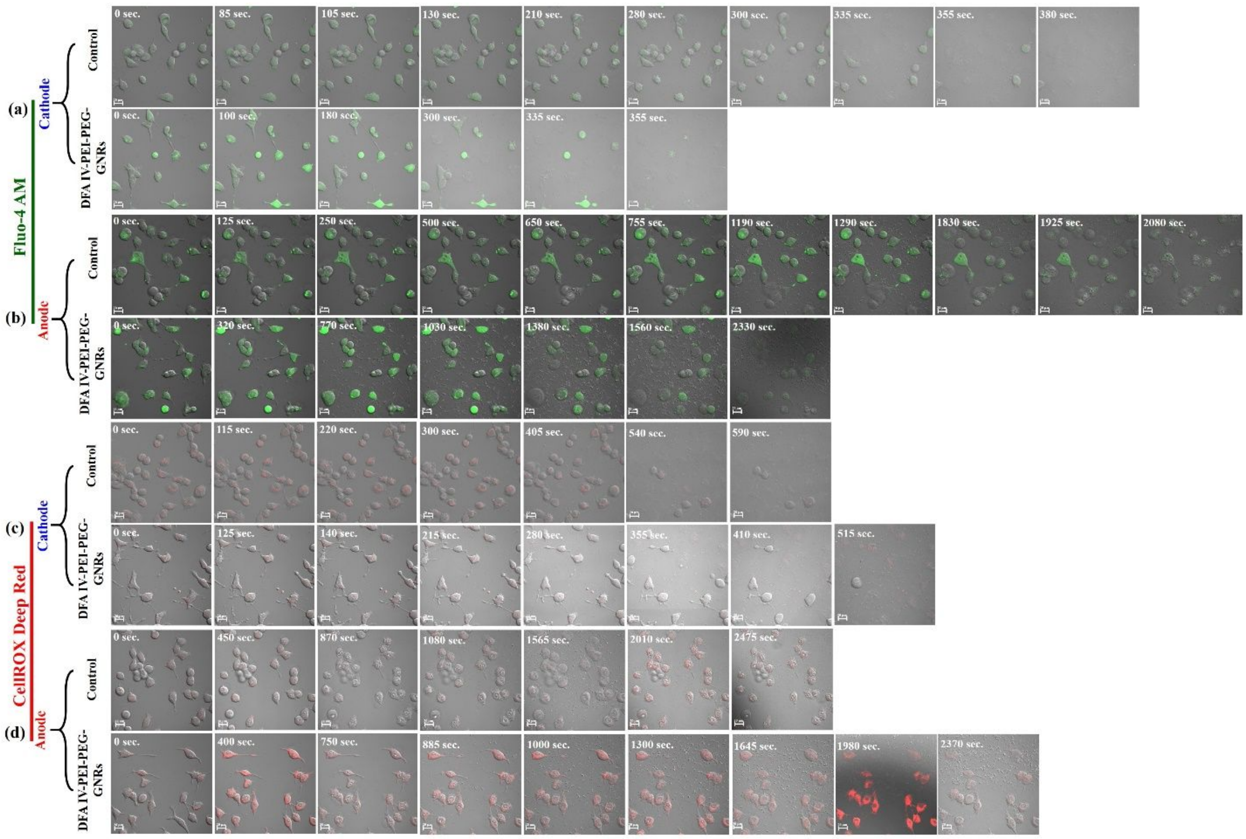

Figure S12. (a-d) Merged images of confocal fluorescence images and bright field images of Ca^{2+} and ROS generated by PBS (control) and DFA IV-PEI-PEG-GNR-treated CT-26 cells at the cathode and anode with and without square-wave DC field for various time intervals using Fluo-4 AM (green) and CellROX Deep Red (ROS) (60× magnification; scale bar: 20 μm).

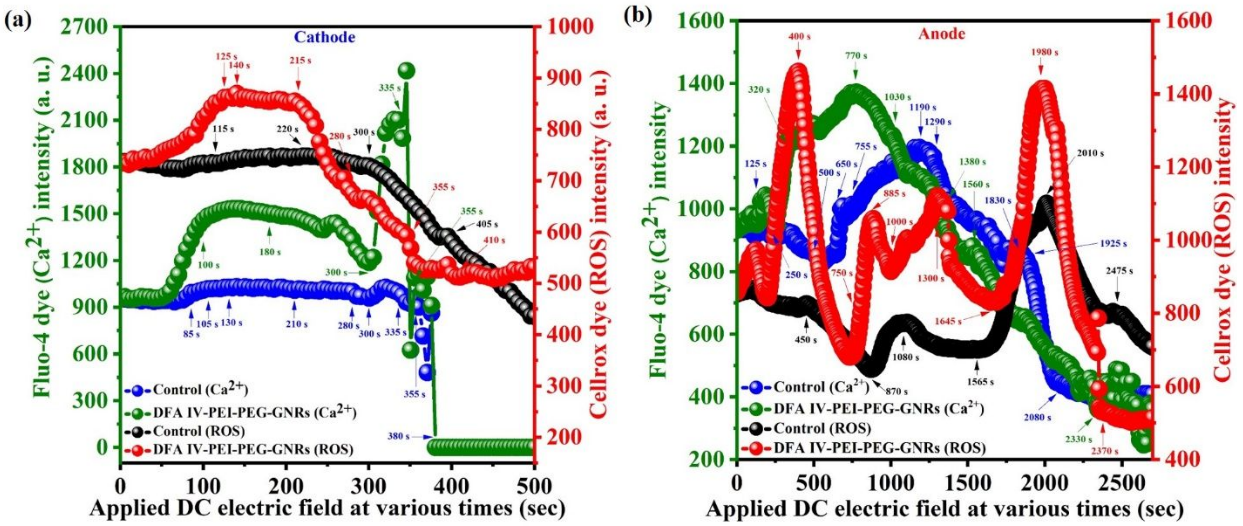

Figure S13. The fluorescence intensities of Ca^{2+} and ROS generated by PBS (control) and DFA IV-PEI-PEG-GNR-treated CT-26 cells at the cathode (a) and anode (b) with/without square-wave DC field for various time intervals using Fluo-4 AM (green) and CellROX Deep Red (ROS).

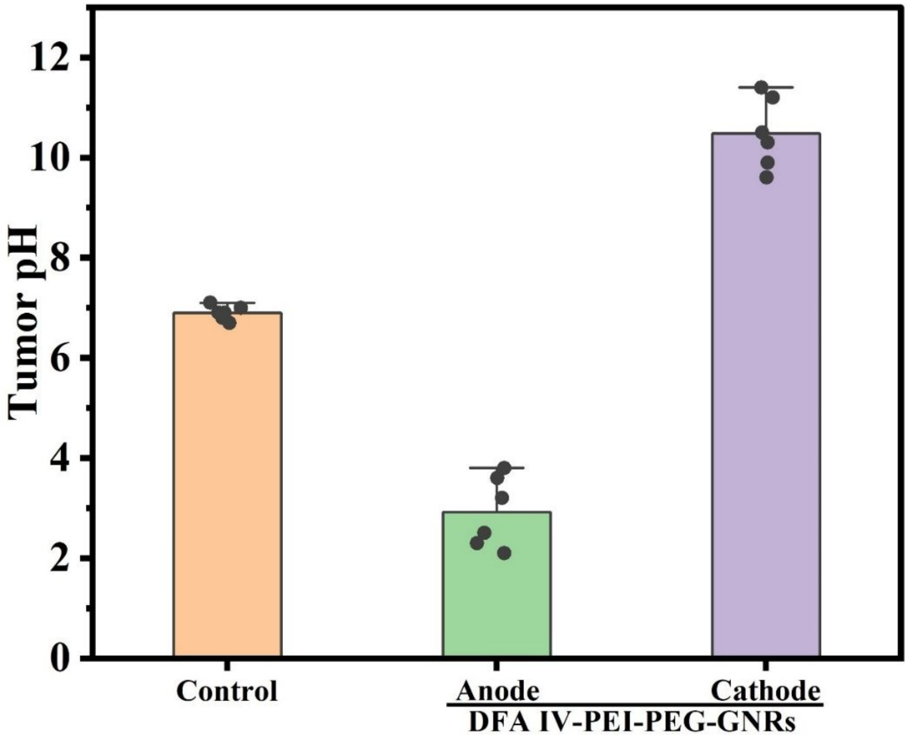

Figure S14. Intratumor pH was measured using a pH electrode with a pH meter connected to two Pt electrodes in response to DFA IV PEI-PEG-GNRs.

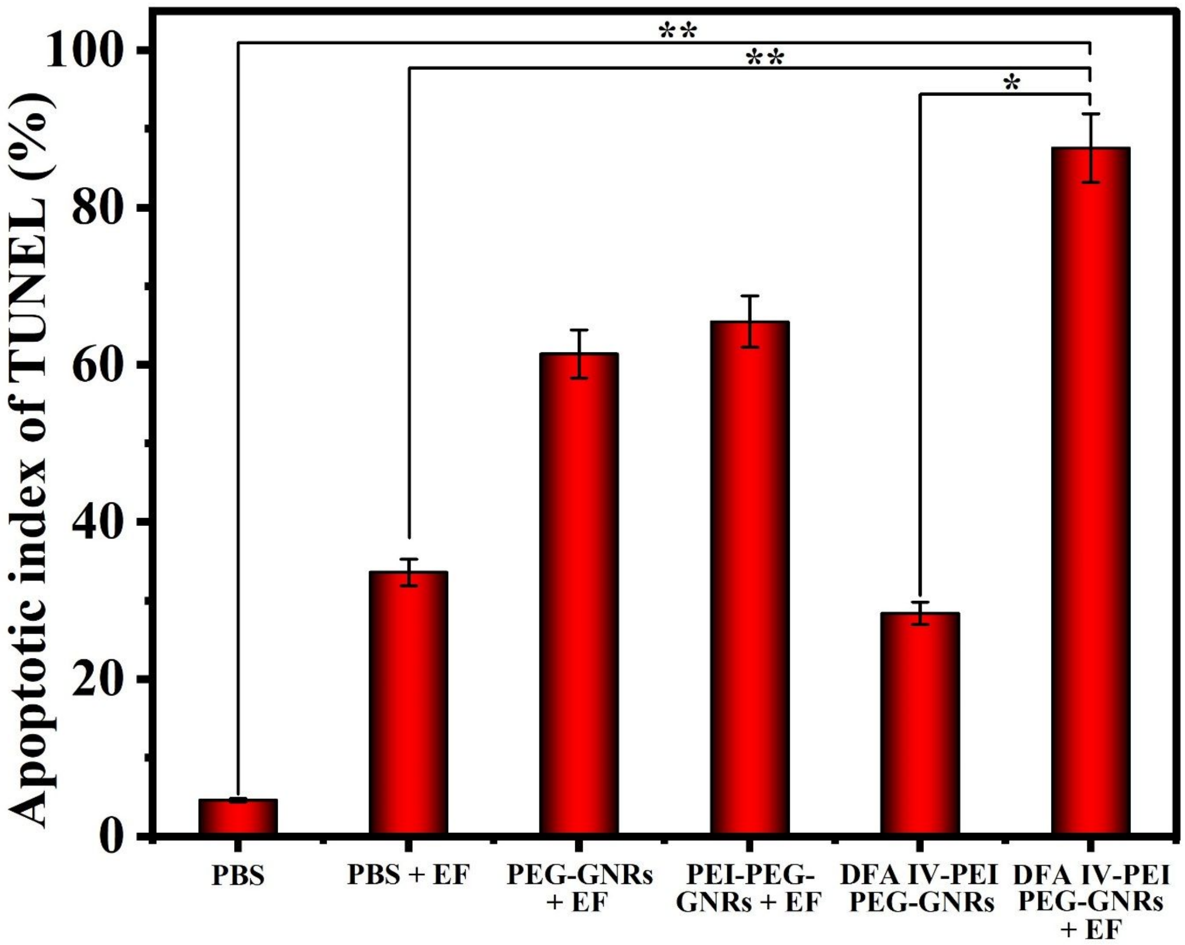

Figure S15. The corresponding apoptotic index of the TUNEL assay in various groups. p values: * $p < 0.05$, ** $p < 0.01$.

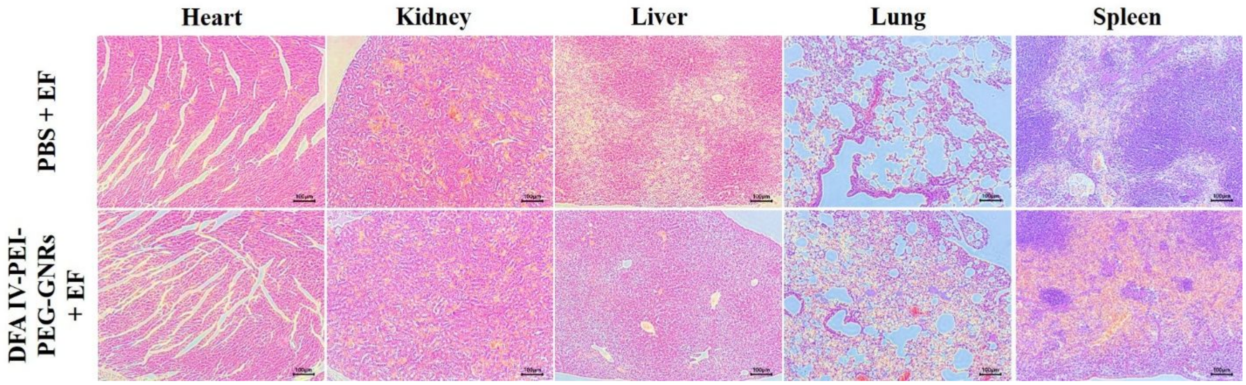

Figure S16. H&E staining of major organs from mice in various groups (10× magnification; scale bar, 100 μm).

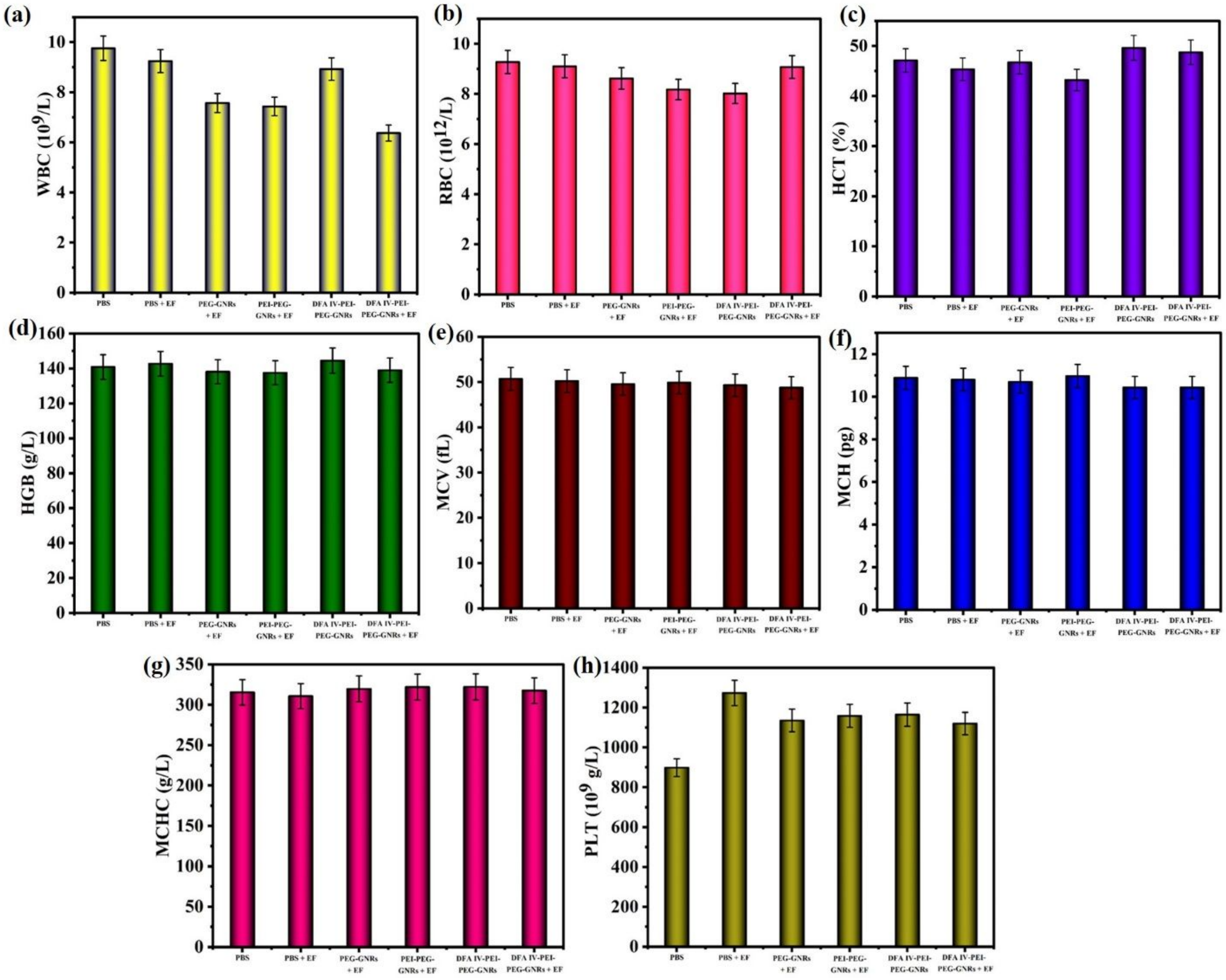

Figure S17. (a-h) Blood biochemical test results after various treatments. The results are presented as the mean and standard deviation of white blood cells (WBC), red blood cells (RBC), hematocrit (HCT), hemoglobin (HGB), mean platelet volume (MCV), mean corpuscular hemoglobin (MCH), mean corpuscular hemoglobin concentration (MCHC), and platelet count (PLT).

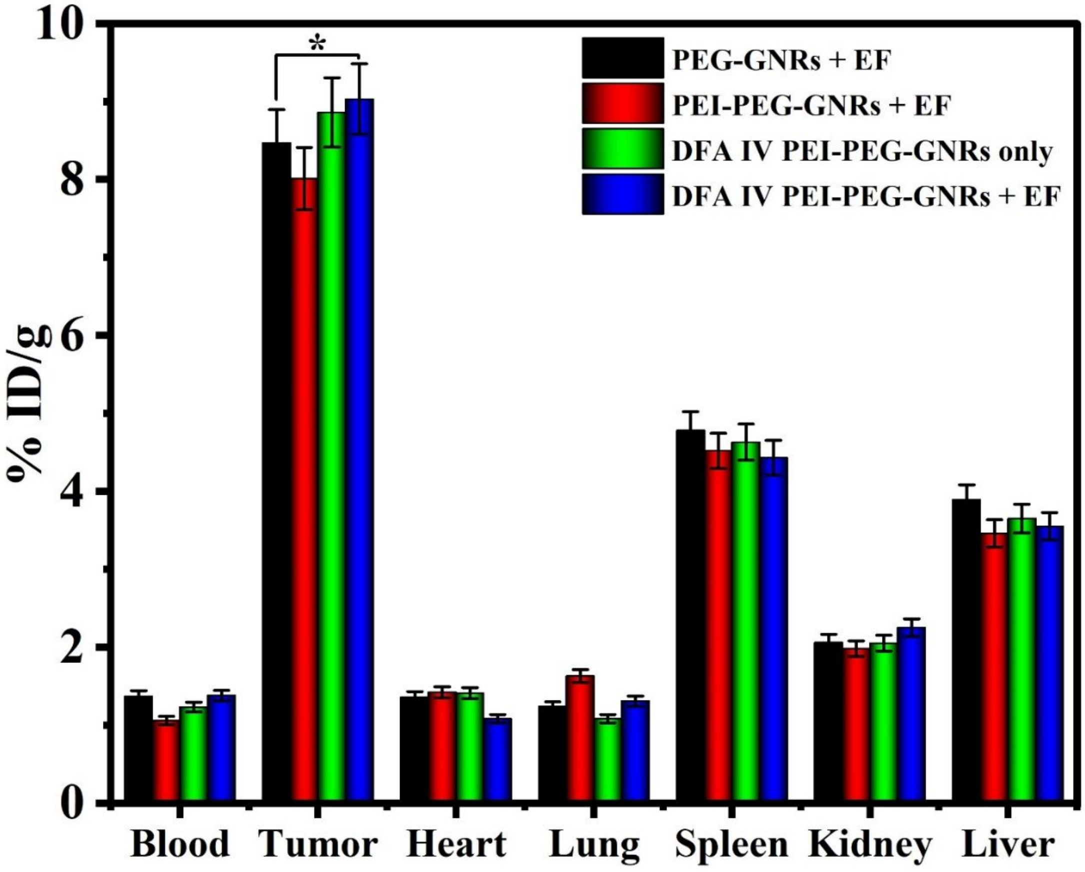

Figure S18. Biodistribution of Au concentrations in the blood, tumors, and major organs of mice treated with various groups.

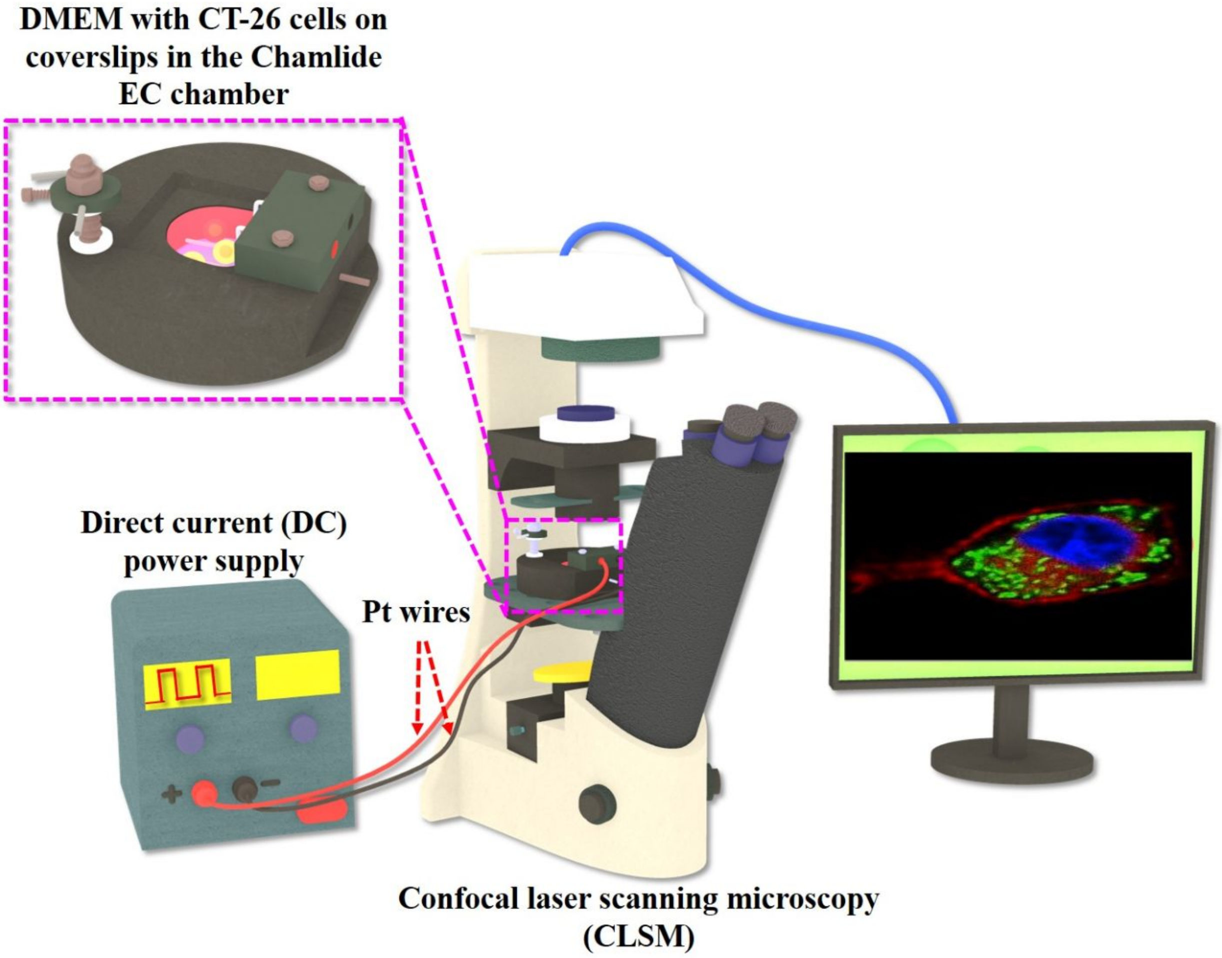

Figure S19. Experimental setup to investigate the therapeutic effects of EA.

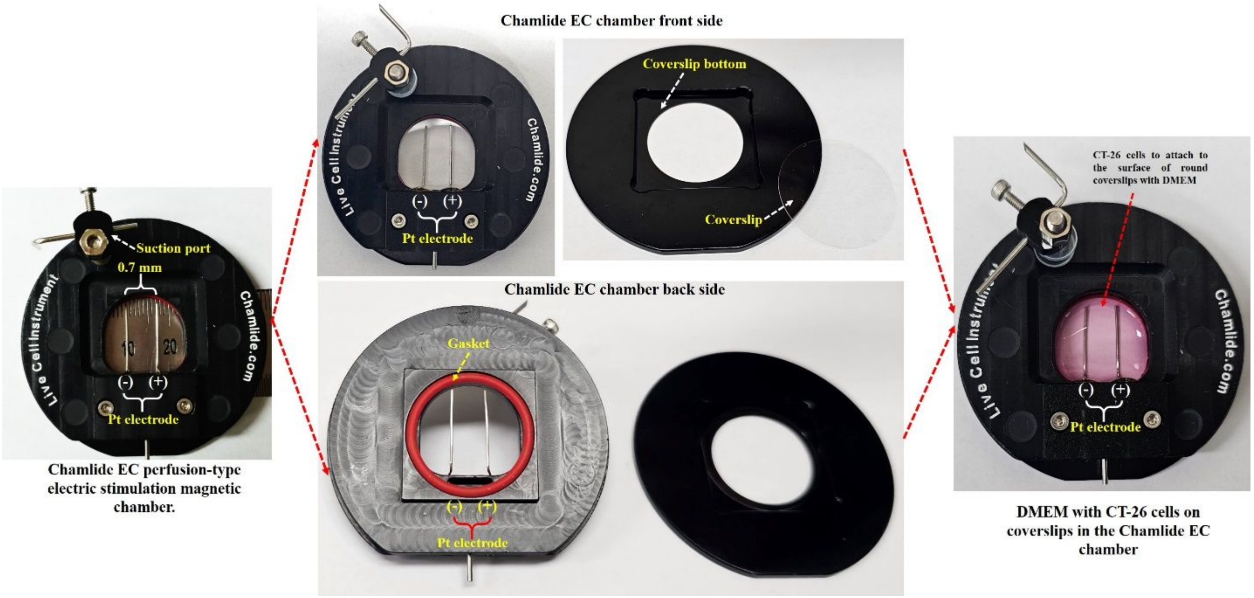

Figure S20. Chamlide EC perfusion-type electric stimulation magnetic chamber with a Pt electrode and suction needle was used to maintain cell culture.

Table S1. Comparative capabilities of recently published cancer treatment approaches based on EA.

Materials	Electrodes	Waveforms	DC output power supply	Duration of DC power	Tested disease	Cancer cell line	Applications	Ref.
DFA IV-PEI-PEG-GNRs	Two Pt electrodes	Square wave	1.0 mA	30 min	Colorectal cancer	CT-26 cells and Male BALB/c nude mice	A more significant reduction in tumor growth rate.	-
Liposic eye gel	Many needle electrodes	-	30 mw	30 min	Breast cancer	4T1 cells and Female inbred BALB/c mice	In the external anode electrochemical therapy group, tumor sizes were significantly decreased.	1
-	Two to five Pt electrodes	-	8.69 mA	114.70 min	Fibrosarcoma	RIF-1 cells, female C3H/HeJ mice, and female Fisher 344 rats	This method significantly increased the destruction rate.	2
-	Two or more Pt electrodes	-	10-1000 mA	30-10 hrs	Mammary cancer	13 Dog's mammary cancer	It is effective in lower-stage tumors.	3
	One to four Pt electrodes	-	80 C/cm^3	24 hrs	Liver metastases of colorectal cancer	DHD/K12 colorectal adenocarcinoma cells and BDIX rats	Tumor necrosis was completely observed in 54% of cases.	4

Supplemental Videos

Video S1. Merged images of confocal fluorescence images and bright field images of CT-26 cells treated with PBS (control) at the cathode under the square-wave DC field for various time intervals where the nuclei, mitochondria, endoplasmic reticulum, and plasma membrane were stained with Hoechst 33342, MitoTracker Green, endoplasmic reticulum (ER)-Tracker Red, and CellMask Deep Red Plasma membrane (60× magnification; scale bar: 10 μm).

Video S2. Merged images of confocal fluorescence images of CT-26 cells treated with PBS (control) at the cathode under the square-wave DC field for various time intervals where the nuclei, mitochondria, endoplasmic reticulum, and plasma membrane were stained with Hoechst 33342, MitoTracker Green, endoplasmic reticulum (ER)-Tracker Red, and CellMask Deep Red Plasma membrane (60× magnification; scale bar: 10 μm).

Video S3. Merged images of confocal fluorescence images and bright field images of CT-26 cells treated with DFA IV-PEI-PEG-GNRs at the cathode under the square-wave DC field for various time intervals where the nuclei, mitochondria, endoplasmic reticulum, and plasma membrane were stained with Hoechst 33342, MitoTracker Green, endoplasmic reticulum (ER)-Tracker Red, and CellMask Deep Red Plasma membrane (60× magnification; scale bar: 10 μm).

Video S4. Merged images of confocal fluorescence images of CT-26 cells treated with DFA IV-PEI-PEG-GNRs at the cathode under the square-wave DC field for various time intervals where the nuclei, mitochondria, endoplasmic reticulum, and plasma membrane were stained with Hoechst 33342, MitoTracker Green, endoplasmic reticulum (ER)-Tracker Red, and CellMask Deep Red Plasma membrane (60× magnification; scale bar: 10 μm).

Video S5. Merged images of confocal fluorescence images and bright field images of CT-26 cells treated with PBS (control) at the middle region under the square-wave DC field for various time intervals where the nuclei, mitochondria, endoplasmic reticulum, and plasma membrane were stained with Hoechst 33342, MitoTracker Green, endoplasmic reticulum (ER)-Tracker Red, and CellMask Deep Red Plasma membrane (60× magnification; scale bar: 10 μm).

Video S6. Merged images of confocal fluorescence images of CT-26 cells treated with PBS (control) at the middle region under the square-wave DC field for various time intervals where the nuclei, mitochondria, endoplasmic reticulum, and plasma membrane were stained with Hoechst 33342, MitoTracker Green, endoplasmic reticulum (ER)-Tracker Red, and CellMask Deep Red Plasma membrane (60× magnification; scale bar: 10 μm).

Video S7. Merged images of confocal fluorescence images and bright field images of CT-26 cells treated with DFA IV-PEI-PEG-GNRs at the middle region under the square-wave DC field for various time intervals where the nuclei, mitochondria, endoplasmic reticulum, and plasma membrane were stained with Hoechst 33342,

MitoTracker Green, endoplasmic reticulum (ER)-Tracker Red, and CellMask Deep Red Plasma membrane (60× magnification; scale bar: 10 μm).

Video S8. Merged images of confocal fluorescence images of CT-26 cells treated with DFA IV-PEI-PEG-GNRs at the middle region under the square-wave DC field for various time intervals where the nuclei, mitochondria, endoplasmic reticulum, and plasma membrane were stained with Hoechst 33342, MitoTracker Green, endoplasmic reticulum (ER)-Tracker Red, and CellMask Deep Red Plasma membrane (60× magnification; scale bar: 10 μm).

Video S9. Merged images of confocal fluorescence images and bright field images of CT-26 cells treated with PBS (control) at the anode under the square-wave DC field for various time intervals where the nuclei, mitochondria, endoplasmic reticulum, and plasma membrane were stained with Hoechst 33342, MitoTracker Green, endoplasmic reticulum (ER)-Tracker Red, and CellMask Deep Red Plasma membrane (60× magnification; scale bar: 10 μm).

Video S10. Merged images of confocal fluorescence images of CT-26 cells treated with PBS (control) at the anode under the square-wave DC field for various time intervals where the nuclei, mitochondria, endoplasmic reticulum, and plasma membrane were stained with Hoechst 33342, MitoTracker Green, endoplasmic reticulum (ER)-Tracker Red, and CellMask Deep Red Plasma membrane (60× magnification; scale bar: 10 μm).

Video S11. Merged images of confocal fluorescence images and bright field images of CT-26 cells treated with DFA IV-PEI-PEG-GNRs at the anode under the square-wave DC field for various time intervals where the nuclei, mitochondria, endoplasmic reticulum, and plasma membrane were stained with Hoechst 33342, MitoTracker Green, endoplasmic reticulum (ER)-Tracker Red, and CellMask Deep Red Plasma membrane (60× magnification; scale bar: 10 μm).

Video S12. Merged images of confocal fluorescence images of CT-26 cells treated with DFA IV-PEI-PEG-GNRs at the anode under the square-wave DC field for various time intervals where the nuclei, mitochondria, endoplasmic reticulum, and plasma membrane were stained with Hoechst 33342, MitoTracker Green, endoplasmic reticulum (ER)-Tracker Red, and CellMask Deep Red Plasma membrane (60× magnification; scale bar: 10 μm).

Video S13. Merged images of confocal fluorescence images and bright field images of DFA IV-PEI-PEG-GNRs-treated CT-26 cells exposed to the strong acidic (pH 2.5) of DMEM for various time intervals without DC treatments where the nuclei, mitochondria, endoplasmic reticulum, and plasma membrane were stained with Hoechst 33342, MitoTracker Green, endoplasmic reticulum (ER)-Tracker Red, and CellMask Deep Red Plasma membrane (60× magnification; scale bar: 10 μm).

Video S14. Merged images of confocal fluorescence images and bright field images of DFA IV-PEI-PEG-GNRs-treated CT-26 cells exposed to the strong acidic (pH 3.4) of DMEM for various time intervals without DC treatments where the nuclei, mitochondria, endoplasmic reticulum, and plasma membrane were stained with Hoechst 33342, MitoTracker Green, endoplasmic reticulum (ER)-Tracker Red, and

CellMask Deep Red Plasma membrane (60× magnification; scale bar: 10 μm).

Video S15. Merged images of confocal fluorescence images and bright field images of DFA IV-PEI-PEG-GNRs-treated CT-26 cells exposed to the strong alkaline (pH 10.3) of DMEM for various time intervals without DC treatments where the nuclei, mitochondria, endoplasmic reticulum, and plasma membrane were stained with Hoechst 33342, MitoTracker Green, endoplasmic reticulum (ER)-Tracker Red, and CellMask Deep Red Plasma membrane (60× magnification; scale bar: 10 μm).

Video S16. Merged images of confocal fluorescence images and bright field images of DFA IV-PEI-PEG-GNRs-treated CT-26 cells exposed to the strong alkaline (pH 11.4) of DMEM for various time intervals without DC treatments where the nuclei, mitochondria, endoplasmic reticulum, and plasma membrane were stained with Hoechst 33342, MitoTracker Green, endoplasmic reticulum (ER)-Tracker Red, and CellMask Deep Red Plasma membrane (60× magnification; scale bar: 10 μm).

Video S17. Merged images of confocal fluorescence images and bright field images of CT-26 cells apoptosis induced by PBS (control) at the cathode with square-wave DC field for various time intervals using the Annexin V-FITC/PI (60× magnification; scale bar: 20 μm).

Video S18. Merged images of confocal fluorescence images and bright field images of CT-26 cells apoptosis induced by DFA IV-PEI-PEG-GNRs at the cathode with square-wave DC field for various time intervals using the Annexin V-FITC/PI (60× magnification; scale bar: 20 μm).

Video S19. Merged images of confocal fluorescence images and bright field images of CT-26 cells apoptosis induced by DFA IV-PEI-PEG-GNRs at the anode with square-wave DC field for various time intervals using the Annexin V-FITC/PI (60× magnification; scale bar: 20 μm).

Video S20. Merged images of confocal fluorescence images and bright field images of CT-26 cells apoptosis induced by PBS (control) at the anode with square-wave DC field for various time intervals using the Annexin V-FITC/PI (60× magnification; scale bar: 20 μm).

Video S21. Merged images of confocal fluorescence images and bright field images of Ca^{2+} generated by DFA IV-PEI-PEG-GNRs-treated CT-26 cells at the cathode with square-wave DC field for various time intervals using Fluo-4 AM (green) (60× magnification; scale bar: 20 μm).

Video S22. Merged images of confocal fluorescence images of Ca^{2+} generated by DFA IV-PEI-PEG-GNRs-treated CT-26 cells at the cathode with square-wave DC field for various time intervals using Fluo-4 AM (green) (60× magnification; scale bar: 20 μm).

Video S23. Merged images of confocal fluorescence images and bright field images of Ca^{2+} generated by DFA IV-PEI-PEG-GNRs-treated CT-26 cells at the anode with square-wave DC field for various time intervals using Fluo-4 AM (green) (60× magnification; scale bar: 20 μm).

Video S24. Merged images of confocal fluorescence images of Ca^{2+} generated by DFA

IV-PEI-PEG-GNRs-treated CT-26 cells at the anode with square-wave DC field for various time intervals using Fluo-4 AM (green) (60× magnification; scale bar: 20 μm).

Video S25. Merged images of confocal fluorescence images and bright field images of Ca^{2+} generated by PBS (control)-treated CT-26 cells at the cathode with square-wave DC field for various time intervals using Fluo-4 AM (green) (60× magnification; scale bar: 20 μm).

Video S26. Merged images of confocal fluorescence images of Ca^{2+} generated by PBS (control)-treated CT-26 cells at the cathode with square-wave DC field for various time intervals using Fluo-4 AM (green) (60× magnification; scale bar: 20 μm).

Video S27. Merged images of confocal fluorescence images and bright field images of Ca^{2+} generated by PBS (control)-treated CT-26 cells at the anode with square-wave DC field for various time intervals using Fluo-4 AM (green) (60× magnification; scale bar: 20 μm).

Video S28. Merged images of confocal fluorescence images of Ca^{2+} generated by PBS (control)-treated CT-26 cells at the anode with square-wave DC field for various time intervals using Fluo-4 AM (green) (60× magnification; scale bar: 20 μm).

Video S29. Merged images of confocal fluorescence images and bright field images of ROS generated by DFA IV-PEI-PEG-GNRs-treated CT-26 cells at the cathode with square-wave DC field for various time intervals using CellROX Deep Red (60× magnification; scale bar: 20 μm).

Video S30. Merged images of confocal fluorescence images of ROS generated by DFA IV-PEI-PEG-GNRs-treated CT-26 cells at the cathode with square-wave DC field for various time intervals using CellROX Deep Red (60× magnification; scale bar: 20 μm).

Video S31. Merged images of confocal fluorescence images and bright field images of ROS generated by DFA IV-PEI-PEG-GNRs-treated CT-26 cells at the anode with square-wave DC field for various time intervals using CellROX Deep Red (60× magnification; scale bar: 20 μm).

Video S32. Merged images of confocal fluorescence images of ROS generated by DFA IV-PEI-PEG-GNRs-treated CT-26 cells at the anode with square-wave DC field for various time intervals using CellROX Deep Red (60× magnification; scale bar: 20 μm).

Video S33. Merged images of confocal fluorescence images and bright field images of ROS generated by PBS (control)-treated CT-26 cells at the cathode with square-wave DC field for various time intervals using CellROX Deep Red (60× magnification; scale bar: 20 μm).

Video S34. Merged images of confocal fluorescence images of ROS generated by PBS (control)-treated CT-26 cells at the cathode with square-wave DC field for various time intervals using CellROX Deep Red (60× magnification; scale bar: 20 μm).

Video S35. Merged images of confocal fluorescence images and bright field images of ROS generated by PBS (control)-treated CT-26 cells at the anode with square-wave DC field for various time intervals using CellROX Deep Red (60× magnification; scale bar: 20 μm).

Video S36. Merged images of confocal fluorescence images of ROS generated by PBS (control)-treated CT-26 cells at the anode with square-wave DC field for various time intervals using CellROX Deep Red (60× magnification; scale bar: 20 μm).

Supplemental References

(1) Miripour, Z. S.; Ghahremani, A.; Karimi, K.; Jahanbakhsh, F.; Abbasvandi, F.; Hoseinpour, P.; Parniani, M.; Abdolahad, M. J. M. O. Electrochemical therapy (EChT) of cancer tumor with an external anode, a way to achieve pathological complete response. **2023**, *40* (4), 117.

(2) Chou, C. K.; McDougall, J. A.; Ahn, C.; Vora, N. Electrochemical treatment of mouse and rat fibrosarcomas with direct current. *Bioelectromagnetics* **1997**, *18* (1), 14-24.

(3) Tello, M.; Oliveira, L.; Parise, O.; Buzaid, A.; Oliveira, R.; Zanella, R.; Cardona, A. Electrochemical therapy to treat cancer (in vivo treatment). In *2007 29th Annual International Conference of the IEEE Engineering in Medicine and Biology Society*, 2007; IEEE: pp 3524-3527.

(4) Maintz, D.; Fischbach, R.; SCHÄFER, N.; SCHÄFER, H.; Gossmann, A.; Kugel, H. Results of electrochemical therapy of colorectal liver metastases in rats followed up by MRI. *Invest. Radiol.* **2000**, *35* (5), 289-294.
